# CRISPR/Cas9 screen uncovers functional translation of cryptic lncRNA-encoded open reading frames in human cancer

**DOI:** 10.1172/JCI159940

**Published:** 2023-03-01

**Authors:** Caishang Zheng, Yanjun Wei, Peng Zhang, Longyong Xu, Zhenzhen Zhang, Kangyu Lin, Jiakai Hou, Xiangdong Lv, Yao Ding, Yulun Chiu, Antrix Jain, Nelufa Islam, Anna Malovannaya, Yun Wu, Feng Ding, Han Xu, Ming Sun, Xi Chen, Yiwen Chen

**Affiliations:** 1 Department of Bioinformatics and Computational Biology, The University of Texas MD Anderson Cancer Center, Houston, Texas, USA.; 2 Department of Molecular and Cellular Biology,; 3 Lester and Sue Smith Breast Center, and; 4 Dan L. Duncan Comprehensive Cancer Center, Baylor College of Medicine, Houston, Texas, USA.; 5 Department of Physics and Astronomy, Clemson University, Clemson, South Carolina, USA.; 6 Department of Melanoma Medical Oncology, The University of Texas MD Anderson Cancer Center, Houston, Texas, USA.; 7 Mass Spectrometry Proteomics Core and; 8 Verna and Marrs McLean Department of Biochemistry and Molecular Biology, Baylor College of Medicine, Houston, Texas, USA.; 9 Department of Pathology, The University of Texas MD Anderson Cancer Center, Houston, Texas, USA.; 10 Department of Epigenetics and Molecular Carcinogenesis, The University of Texas MD Anderson Cancer Center,; 11 Genetics and Epigenetics Program, and; 12 Quantitative Sciences Program, MD Anderson Cancer Center UTHealth Graduate School of Biomedical Sciences, Houston, Texas, USA.

**Keywords:** Genetics, Oncology, Breast cancer, Noncoding RNAs, Translation

## Abstract

Emerging evidence suggests that cryptic translation within long noncoding RNAs (lncRNAs) may produce novel proteins with important developmental/physiological functions. However, the role of this cryptic translation in complex diseases (e.g., cancer) remains elusive. Here, we applied an integrative strategy combining ribosome profiling and CRISPR/Cas9 screening with large-scale analysis of molecular/clinical data for breast cancer (BC) and identified estrogen receptor α–positive (ER^+^) BC dependency on the cryptic ORFs encoded by lncRNA genes that were upregulated in luminal tumors. We confirmed the in vivo tumor-promoting function of an unannotated protein, GATA3-interacting cryptic protein (GT3-INCP) encoded by *LINC00992*, the expression of which was associated with poor prognosis in luminal tumors. GTE-INCP was upregulated by estrogen/ER and regulated estrogen-dependent cell growth. Mechanistically, GT3-INCP interacted with GATA3, a master transcription factor key to mammary gland development/BC cell proliferation, and coregulated a gene expression program that involved many BC susceptibility/risk genes and impacted estrogen response/cell proliferation. GT3-INCP/GATA3 bound to common *cis* regulatory elements and upregulated the expression of the tumor-promoting and estrogen-regulated BC susceptibility/risk genes *MYB* and *PDZK1*. Our study indicates that cryptic lncRNA-encoded proteins can be an important integrated component of the master transcriptional regulatory network driving aberrant transcription in cancer, and suggests that the “hidden” lncRNA-encoded proteome might be a new space for therapeutic target discovery.

## Graphical abstract

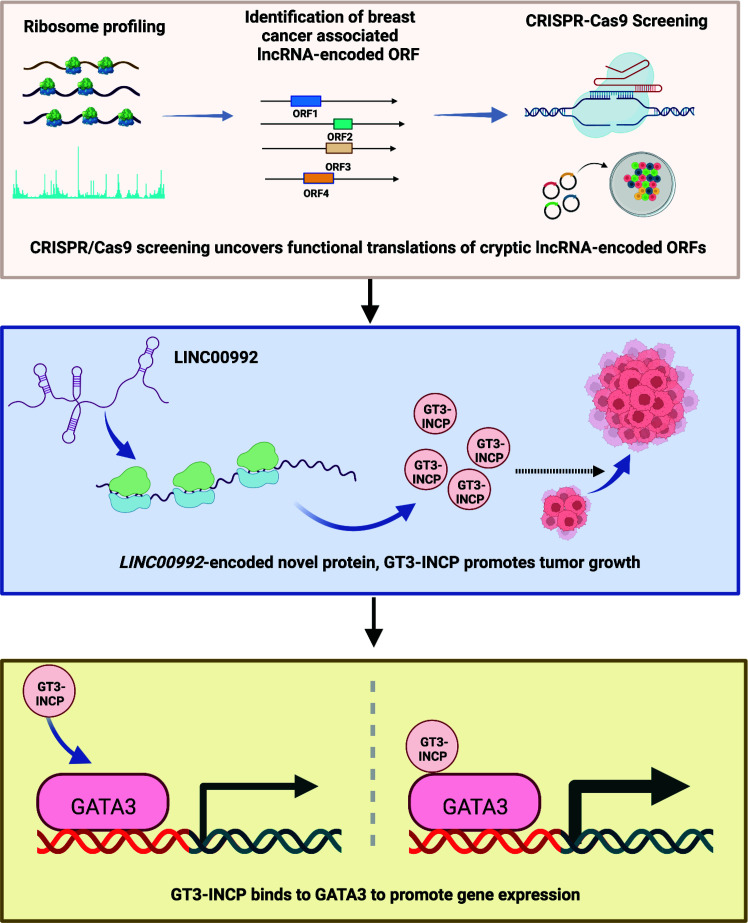

## Introduction

Recent effort from the Encyclopedia of DNA Elements (ENCODE)/GENCODE (1, 2) project has revealed a pervasive transcription of over 70% of the human genome that produces a complex repertoire of transcripts, including both short (<200 nt) and long ones (≥200 nt) (3). Many long transcripts in the human transcriptome show little or no protein-coding capacity based on sequence-based computational analyses and are thus called long noncoding RNAs (lncRNAs). Systematic efforts to catalog lncRNAs using epigenomic or transcriptome data identified more than 15,000 lncRNA genes in the human genome (2, 4). Compared with protein-coding transcripts, lncRNAs tend to be shorter, have fewer exons, and exhibit more tissue-restricted expression (4, 5). lncRNAs are an emerging class of regulatory RNAs that exert diverse functions in different biological processes (6–8). The discovery of lncRNAs in unicellular eukaryotes (9) suggests that their regulatory role may be ancient and beyond multicellular organisms. Moreover, there is mounting evidence that lncRNAs can play an important role in human cancer by promoting cellular pathways that lead to tumorigenesis or tumor suppression (10, 11).

Although lncRNAs are traditionally considered not coding for proteins, a growing body of evidence supports the notion that a fraction of lncRNAs undergo active translation and encode cryptic proteins (12–16). Given their shorter length compared with the annotated protein-coding RNAs, the lncRNA-encoded proteins are usually smaller than the annotated ones. Microproteins (also termed micropeptides, <100 amino acids) produced by cryptic translation within lncRNAs have been shown to play important developmental and physiological roles in evolutionarily distant species (17–21). Despite an increasing appreciation of the functional importance of cryptic lncRNA-encoded proteins in development and physiology, their functional role and molecular mechanism in complex diseases such as cancer remain poorly understood.

To fill this gap, we devised an integrative functional genomic strategy combining ribosome profiling (ribo-seq) (22), a technique that enables the high-resolution measurement of translation on a genome-wide scale, and CRISPR/Cas9 knockout screening (23) with large-scale computational analysis of The Cancer Genome Atlas (TCGA) (24) data, which enables a systematic identification of the human cancer dependency on cryptic lncRNA-encoded ORFs. As a proof-of-principle study, we applied this integrative genomic strategy to breast cancer (BC), the most common cancer (besides skin cancer) and one of the leading causes of cancer-related death in women, to uncover the cryptic lncRNA-encoded proteins that may be functionally important and potentially clinically relevant in estrogen receptor α–positive (ER^+^) BC that accounts for more than two-thirds of all BC cases.

We further characterized the function and mechanism of a cryptic *LINC00992*-encoded protein that was identified from the CRISPR screen and interacted with GATA3. This protein was thus named GATA3-interacting cryptic protein (GT3-INCP).

## Results

### An integrative functional genomic strategy for identifying ER^+^ BC dependency on cryptic lncRNA-encoded ORFs.

**Figure 1:**
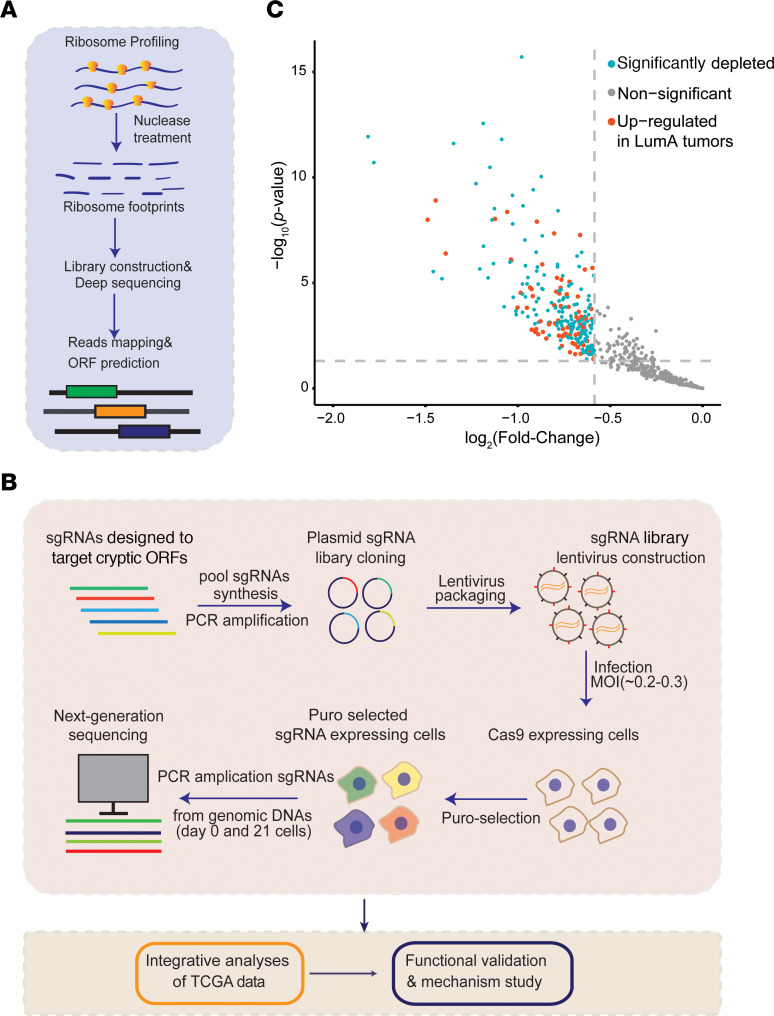
An integrative functional genomic strategy for identifying ER^+^ breast cancer dependency on cryptic lncRNA-encoded ORFs. Workflow diagram depicting the integrative strategy for (**A**) predicting cryptic lncRNA-encoded ORFs from ribo-seq data and (**B**) identifying ER^+^ BC dependency on these predicted cryptic ORFs by using the CRISPR/Cas9 screen. (**C**) Scatter plot showing the statistical significance [–log_10_(*P* value)] and the magnitude of change [log_2_(fold change)] between day 21 and day 0, for the representative negatively selected sgRNAs of the corresponding ORFs. The blue dots correspond to the cryptic lncRNA-encoded ORFs with at least 1 significantly and negatively selected targeting sgRNA in the CRISPR/Cas9 screen and the red dots correspond to the ORFs meeting the criterion of the blue dots, whose host lncRNA expression was significantly upregulated in luminal A (LumA) BC in comparison with the corresponding normal breast tissues, based on TCGA RNA-seq data. Puro, puromycion.

To systematically identify the cryptic lncRNA-encoded ORFs undergoing active translation in ER^+^ BC, we first performed ribo-seq, as described previously (25), to map the translatome in MCF7, an ER^+^ luminal BC cell line (Figure 1A). Quality control of the ribo-seq data (see Supplemental Methods; supplemental material available online with this article; https://doi.org/10.1172/JCI159940DS1) showed that a typical distribution of ribosome-protected fragment (RPF) length peaked around 30 nt (Supplemental Figure 1A). In addition, there was a notable subcodon phasing or 3-nt periodicity of the RPF count across 3 reading frames, an increase in RPF count near annotated translation initiation sites, and a reduction near annotated translation termination sites (Supplemental Figure 1A). The gene-level RPF count from the 3 replicates also showed a significant correlation (Pearson’s *r* > 0.95, *P* < 2.2 × 10^–16^) with each other (Supplemental Figure 1B). These results suggested good quality of the ribo-seq data. We then used Ribo-TISH (25) to predict the cryptic lncRNA-encoded ORFs that may undergo active translation from both in-house and publicly available ribo-seq data generated in MCF7 cells (see Supplemental Methods). We focused on 758 cryptic lncRNA-encoded ORFs with ATG start codons that were identified by Ribo-TISH for further functional genomic study.

To systematically identify the cryptic lncRNA-encoded ORFs that may produce functional proteins and critically contribute to cell growth and/or survival (fitness), we conducted a CRISPR/Cas9-based pooled knockout screen (Figure 1B). We first designed and generated a pooled CRISPR single guide RNA (sgRNA) library (Figure 1A and Supplemental Methods) that contained 3,913 sgRNAs targeting the cryptic ORFs identified from ribo-seq data in MCF7 cells, as well as 636 positive and 1,064 negative control sgRNAs (Supplemental Figure 1C and Supplemental Methods). The screen was then conducted in MCF7 cells that stably expressed wild-type *Streptococcus* *pyogenes* Cas9, in a similar way to our previous study (26). Briefly, the MCF7 cells transduced with the lentiviral vectors encoding the sgRNA library were selected with puromycin. The puromycin-selected cells were passaged for 21 days. The abundance change in individual sgRNAs between the cells collected on day 0 and day 21 was quantified by next-generation sequencing to identify the ORFs that are critical for cell fitness (Supplemental Methods). As expected for the working positive controls, we observed a notable depletion in the abundance of the sgRNAs targeting positive control core essential genes in final cell populations (day 21) compared with the initial (day 0) ones (Supplemental Figure 1D).

**Figure 2:**
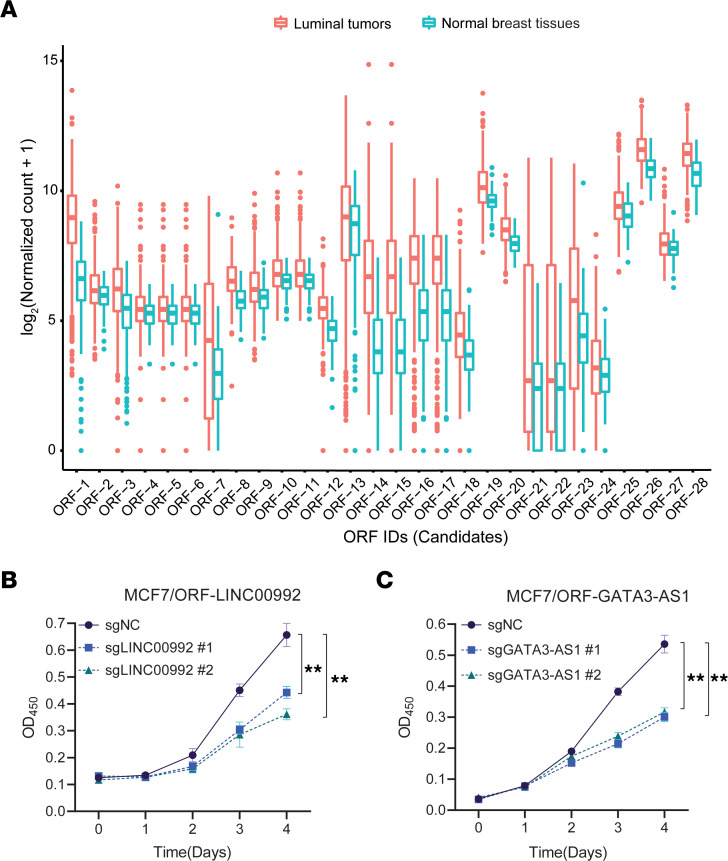
RNA-seq–based expression of the lncRNA genes encoding the screen hits in tumors and normal tissues from TCGA and validation of the hits encoded by LINC00992/GATA3-AS1. (**A**) Box-and-whisker plot showing the expression of the corresponding lncRNA genes that encode the 28 screen hits of cryptic ORFs and were upregulated in luminal BC tumors with respect to the normal breast tissues, based on TCGA data. The bottom and top edges of the box represent the lower and upper quartiles. The median marks the midpoint of the data and is shown by the line dividing the box into 2 parts. The whiskers represent the values between the bottom 5% and 25% or between the top 25% and 5%. The outliers are shown as points. The growth of MCF7 cells transduced with negative control sgRNA (sgNC) or gene-specific sgRNAs targeting (**B**) ORF-LINC00992 or (**C**) ORF-GATA3-AS1 was monitored via CCK-8 assay. The OD_450_ for the water-soluble tetrazolium 8 (WST-8) product formazan was measured each day for 4 days via CCK-8 assay. Data in **B** and **C** are shown as mean ± SD (*n* = 3). **P < 0.01 by 1-way ANOVA with Dunnett’s multiple-comparison test. NS, not significant (*P* > 0.05).

By integrative analysis of CRISPR screen data and The Cancer Genome Atlas (TCGA) (27), RNA-seq data in luminal (28) BC and normal breast tissues (Supplemental Methods), and removing highly similar ORFs, we identified 28 cryptic ORFs (Supplemental Table 1) that had at least 2 significantly negatively selected targeting sgRNAs (log_2_[fold change] ≤ –log_2_[1.5], *P* < 0.05). These ORFs were encoded by lncRNA genes whose RNA expression was significantly upregulated in luminal A BC compared with normal breast tissues (log_2_[fold change] ≥ log_2_[1.2], FDR < 0.01; Figures 1C and 2A), and represent the candidates that may be clinically relevant in ER^+^ BC. We further performed functional validation of the 2 cryptic ORF hits that are encoded by the lncRNA gene *LINC00992* (ORF7) and *GATA3-AS1* (ORF1). *LINC00992* RNA expression showed a significant association with patient overall survival (log-rank test, *P* = 0.011) in luminal tumors. To validate their functional role in promoting ER^+^ BC cell growth, we selected the top 2 sgRNAs that showed the strongest growth inhibitory effect in our CRISPR screen for each ORF in a loss-of-function study. Consistent with our CRISPR screen results, sgRNA-mediated knockout of ORF-LINC00992/ORF-GATA3-AS1 inhibited the growth of MCF7 (Figure 2, B and C) and T47D cells (Supplemental Figure 1, E and F). In addition, sgRNA-mediated knockout of ORF-LINC00992/ORF-GATA3-AS1 impaired the clonogenic capacity of MCF7 (Supplemental Figure 1, G and H) and T47D cells (Supplemental Figure 1, I and J). We found that overexpressing the wild-type ORF-GATA3-AS1 (Supplemental Figure 1K), which is inherently resistant to siRNAs targeting the regions outside coding sequence (CDS) of GATA3-AS1 RNA, rescued the growth defect caused by siRNA-mediated GATA3-AS1 knockdown (Supplemental Figure 1, L–N), whereas overexpressing the mutant ORF with an ATG-to-AGG mutation in the start codon that abolished protein production (Supplemental Figure 1K) failed to do so (Supplemental Figure 1, M and N). Similarly, overexpression of the wild-type ORF-GATA3-AS1, but not the AGG mutant, rescued the clonogenicity defect caused by GATA3-AS1 knockdown (Supplemental Figure 1, O and P).

### LINC00992 encodes an unannotated protein.

**Figure 3:**
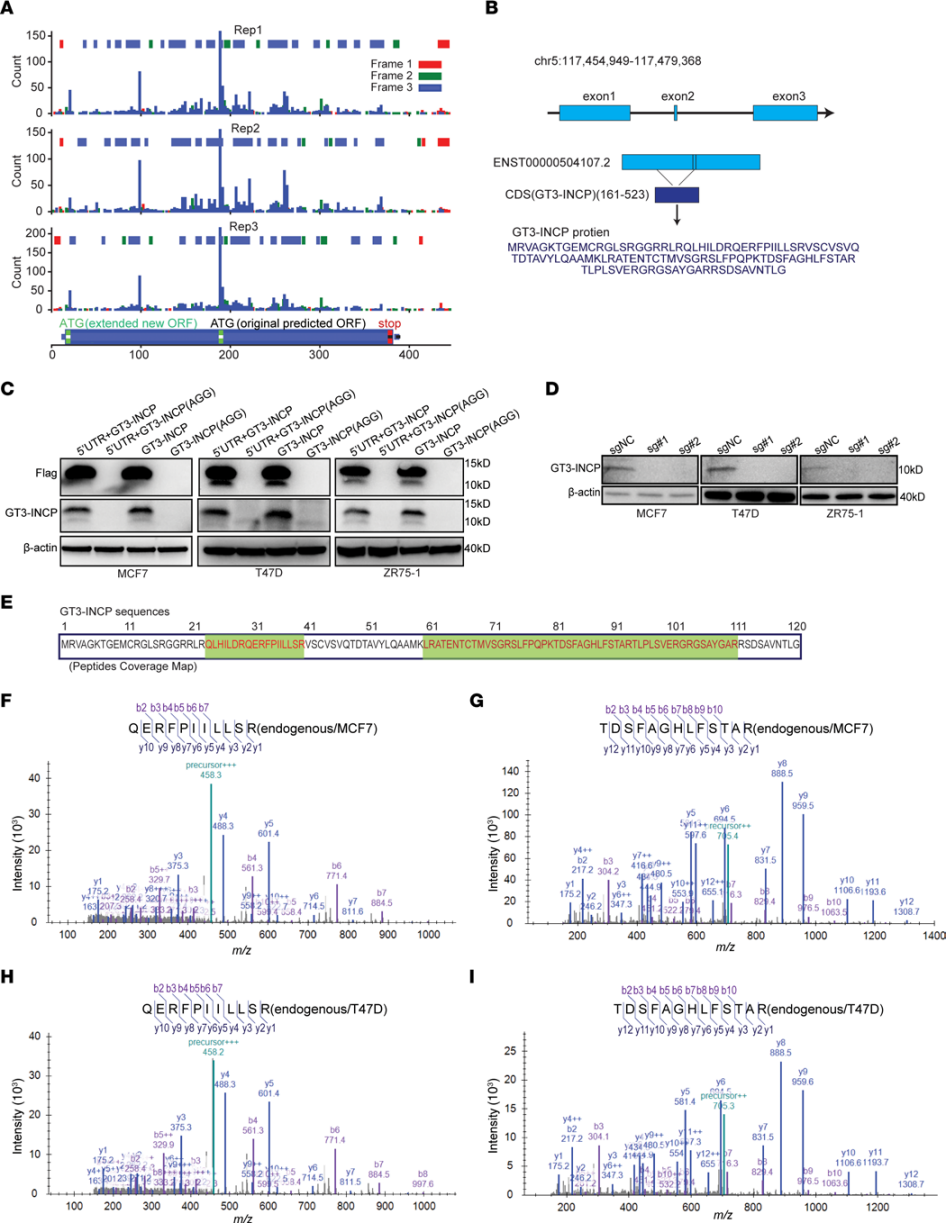
*LINC00992* encodes an unannotated protein. (**A**) Ribo-seq count profile of 3 replicates across the *LINC00992*-encoded ORF. The predicted ORF based on GENCODE v22 annotation (ENST00000504107.1) is labeled “original predicted ORF” and the ORF with the extended region identified by 5′ RACE is labeled “extended new ORF.” (**B**) Schematic of *LINC00992* gene and transcript (ENST00000504107.2, GENCODE v39) structure, and the information about its encoded protein GT3-INCP. (**C**) In the presence/absence of the native 5′-UTR, the wild-type FLAG-tagged GT3-INCP or the mutant one (AGG mutation in start codon) was stably expressed in MCF7, T47D, and ZR75-1 cells and protein expression was determined by Western blot with anti-FLAG and anti–GT3-INCP antibodies, where β-actin was used as a loading control. (**D**) Endogenous GT3-INCP protein expression was determined by Western blot in the indicated ER^+^ BC cell lines that were transduced with the negative control sgRNA (sgNC) or gene-specific sgRNAs, where β-actin served as a loading control. (**E**) The regions of GT3-INCP with the MS-identified peptides from IP of both ectopic FLAG-tagged and endogenous GT3-INCP in ER^+^ BC cells are shown in green and the corresponding sequences are shown in red. (**F**–**I**) The MS2 spectra of the GT3-INCP–derived tryptic peptides QERFPIILLSR and TDSFAGHLFSTAR detected by PRM-MS in the proteins coimmunoprecipitated with the anti-FLAG antibody from MCF7 (**F** and **G**) and T47D (**H** and **I**) cell lysates. Data in **C** and **D** are representative of 3 independent experiments.

Given the significant association between *LINC00992* RNA expression and poor prognosis in luminal BC (Supplemental Figure 2A), we focused on its encoded cryptic ORF for further investigation. *LINC00992* is an intergenic lncRNA gene located on chromosome 5q23.1. To date, there have been no studies to our knowledge indicating that *LINC00992* encodes a protein and that its function is coding dependent. We first performed 5′ and 3′ rapid amplification of cDNA ends (RACE) to verify the full-length *LINC00992* transcript (GENCODE v22 ENST00000504107.1; https://www.gencodegenes.org/) (Supplemental Figure 2B and Supplemental Methods). Although the 3′ end was the same as the original GENCODE v22 transcript annotation, we identified an extension of the 5′ end of the *LINC00992* transcript (Supplemental Figure 2B). Consistently, a strong ribo-seq signal was observed and suggested active translation in the extended region (Figure 3A). The discovery of this extended region was supported by the latest GENCODE v39 transcript annotation (ENST00000504107.2) (Figure 3B). A rabbit polyclonal antibody was developed (Supplemental Methods) and was able to detect the polypeptide produced by the ectopically expressed FLAG-tagged CDS corresponding to GT3-INCP in the absence/presence of its native 5′-UTR in Western blots, whereas the AGG start codon mutation abolished the protein translation and signal in Western blots (Figure 3C). This antibody was also able to detect the endogenous GT3-INCP in ER^+^ BC cells, and the sgRNA-mediated knockout of GT3-INCP reduced the Western blot signal (Figure 3D), suggesting a high specificity of this antibody.

We further confirmed the ectopic protein expression of FLAG-tagged GT3-INCP in the presence of its native 5′-UTR by detecting its constituent peptides with mass spectrometry (MS) (Figure 3E and Supplemental Figure 2, C and D) in the immunoprecipitation (IP) samples generated by an anti-FLAG antibody from the ER^+^ BC cells stably expressing FLAG-tagged GT3-INCP (see Methods). To validate the endogenous protein expression of GT3-INCP, we performed parallel reaction-monitoring MS (PRM-MS) (29) in the IP samples generated by an anti–GT3-INCP antibody from ER^+^ BC cells (see Methods). The PRM-MS data identified the constituent peptides of GT3-INCP and supported its endogenous expression in ER^+^ BC cells (Figure 3, E–I, Supplemental Figure 2, E–H, and Supplemental Table 2). To determine the subcellular localization of GT3-INCP, we performed immunofluorescent staining in the BC cells stably expressing FLAG-tagged GT3-INCP with an anti-FLAG antibody as well as subcellular fractionation followed by Western blotting with an anti–GT3-INCP antibody in ER^+^ BC cells. We found that GT3-INCP was localized in both the nucleus and cytoplasm (Supplemental Figure 2, I and J).

### GT3-INCP is upregulated in ER^+^ tumors and exerts a tumor-promoting function.

**Figure 4:**
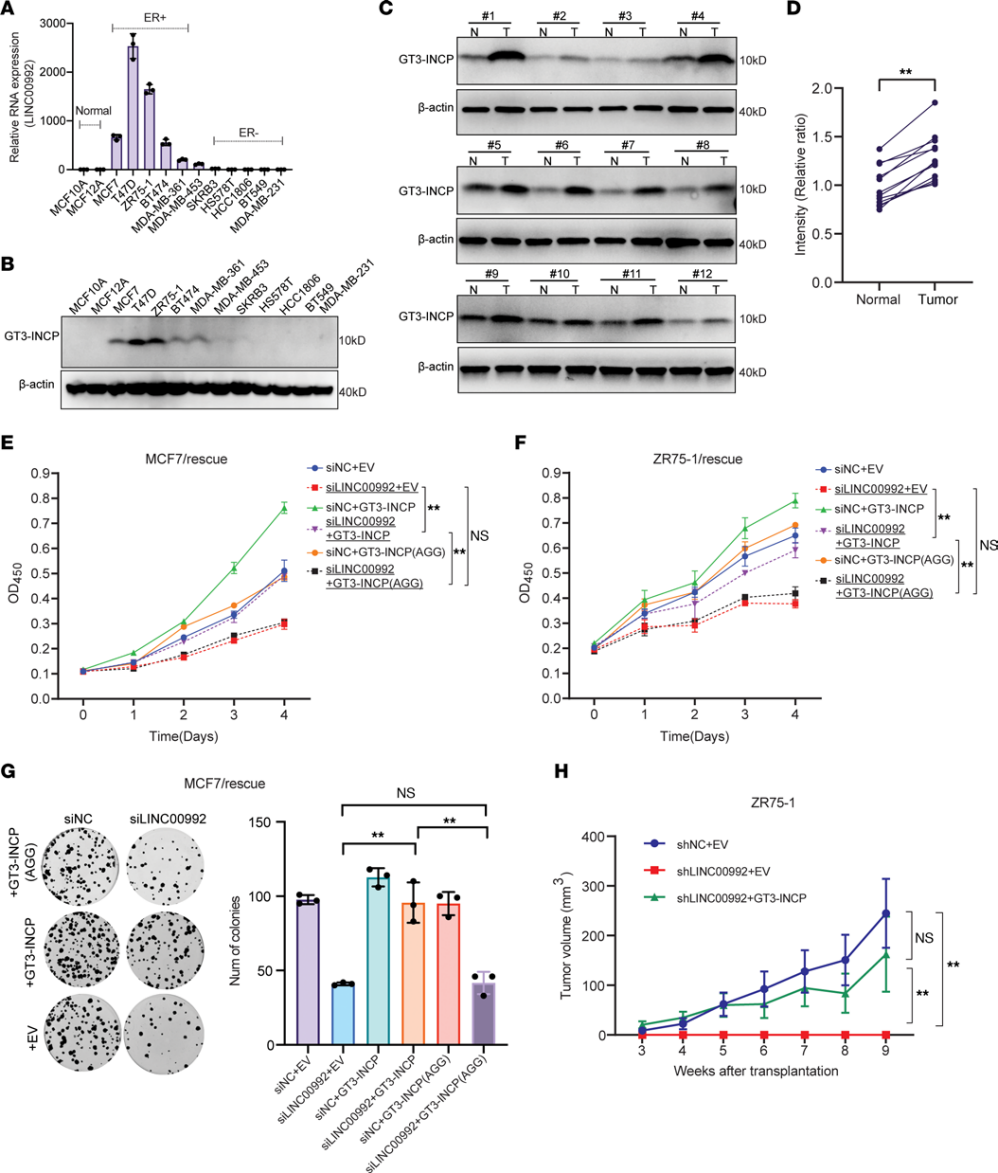
GT3-INCP is upregulated in ER^+^ tumors and exerts a tumor-promoting function. (**A**) qRT-PCR analysis of *LINC00992* RNA expression (*n* = 3) and (**B**) Western blot analysis of GT3-INCP expression in the indicated breast epithelial cells and BC cell lines. For qRT-PCR analysis, *GAPDH* served as an internal control and all expression was relative to that in MCF10A cells. For Western blot analysis, β-actin served as an internal control. (**C**) Western blot analysis of GT3-INCP expression in ER^+^ luminal tumors (T) and the matched normal (N) breast tissue (*n* = 12). (**D**) The GT3-INCP protein level relative to that of β-actin was quantified by densitometry and plotted. (**E**) MCF7 and (**F**) ZR75-1 cells stably transduced with GT3-INCP that has a wild-type (ATG) or mutant (AGG) start codon or the empty vector (EV) control were transfected with the negative control siRNA (siNC) or LINC00992-targeting siRNAs. Cell growth was monitored for 4 days via CCK-8 assay. (**G**) Representative pictures of clonogenic growth and a bar graph quantifying the colonies formed by the MCF7 cells that were transduced with wild-type or mutant (AGG start codon) GT3-INCP or the EV control and were transfected with siNC or siRNAs targeting LINC00992. (**H**) Volume of the orthotopic tumors formed by the ZR75-1 cells that were stably transduced with 3 different combinations (*n* = 6 per combination): EV and shNC, EV and shLINC00992, or GT3-INCP and shLINC00992, was monitored as indicated in the Methods. Data are shown as mean ± SD; *n* = 3 (**E**–**G**) or *n* = 6 (**H**). **P < 0.01 by 2-tailed, paired Student’s *t* test (**D**) or 1-way ANOVA with Tukey’s multiple-comparison test (**E**–**H**). NS, not significant (*P* > 0.05). Data in **B** and **C** are representative of 3 independent experiments.

We performed real-time quantitative reverse transcription PCR (qRT-PCR) and Western blotting in 5 ER^+^ and 6 ER^–^ BC cell lines along with 2 breast epithelial cell lines that are commonly used as a normal breast cell model to determine the difference in the expression of *LINC00992* RNA and GT3-INCP protein across these cell lines. Consistent with our finding that *LINC00992* RNA expression is upregulated in luminal tumors based on TCGA RNA-seq data, the expression of *LINC00992* RNA and GT3-INCP protein was elevated in ER^+^ BC cell lines compared with ER^–^ BC and breast epithelial cell lines (Figure 4, A and B). Moreover, Western blot analysis of fresh-frozen ER^+^ tumors and matched normal breast tissues confirmed that GT3-INCP was upregulated at the protein level in ER^+^ tumors (Figure 4, C and D).

To validate the tumor-promoting function of GT3-INCP with an alternative approach to CRISPR/Cas9, we designed 2 siRNAs targeting the regions outside the CDS in *LINC00992* RNA so that the ectopic expression of GT3-INCP would not be affected by these siRNAs for a loss-of-function study. We found that effective siRNA-mediated knockdown of LINC00992 (Supplemental Figure 3, A–C) inhibited the growth (Supplemental Figure 3, D–F) and impaired the clonogenic capacity of ER^+^ BC cells (Supplemental Figure 3, G and H). Overexpression of GT3-INCP that is inherently resistant to the siRNAs (Supplemental Figure 3, I–K) rescued the growth and clonogenicity defect caused by LINC00992 knockdown (Figure 4, E–G, and Supplemental Figure 3, L and M), whereas overexpression of the mutant GT3-INCP with the AGG mutation in the start codon that abolished protein production (Supplemental Figure 3, I–K) failed to do so (Figure 4, E–G, and Supplemental Figure 3, L and M), supporting the hypothesis that the ORF function is coding dependent. These results further supported the growth-promoting function of GT3-INCP in vitro. To confirm the function of GT3-INCP in vivo, we characterized the loss-of-function phenotype of shRNA-mediated LINC00992 knockdown and the rescue effect of overexpressing GT3-INCP on the shRNA-mediated phenotype in an orthotropic xenograft tumor model (see Methods). We found that in comparison with the negative control shRNA (shNC), shRNA-mediated LINC00992 knockdown abolished the tumor formation in vivo (Figure 4H). Importantly, overexpression of GT3-INCP rescued the shRNA-mediated defects in tumor formation in vivo (Figure 4H). Taken together, these results demonstrated that GT3-INCP exerted a tumor-promoting function both in vitro and in vivo.

### GT3-INCP interacts with GATA3 and this interaction is important for GT3-INCP function.

**Figure 5:**
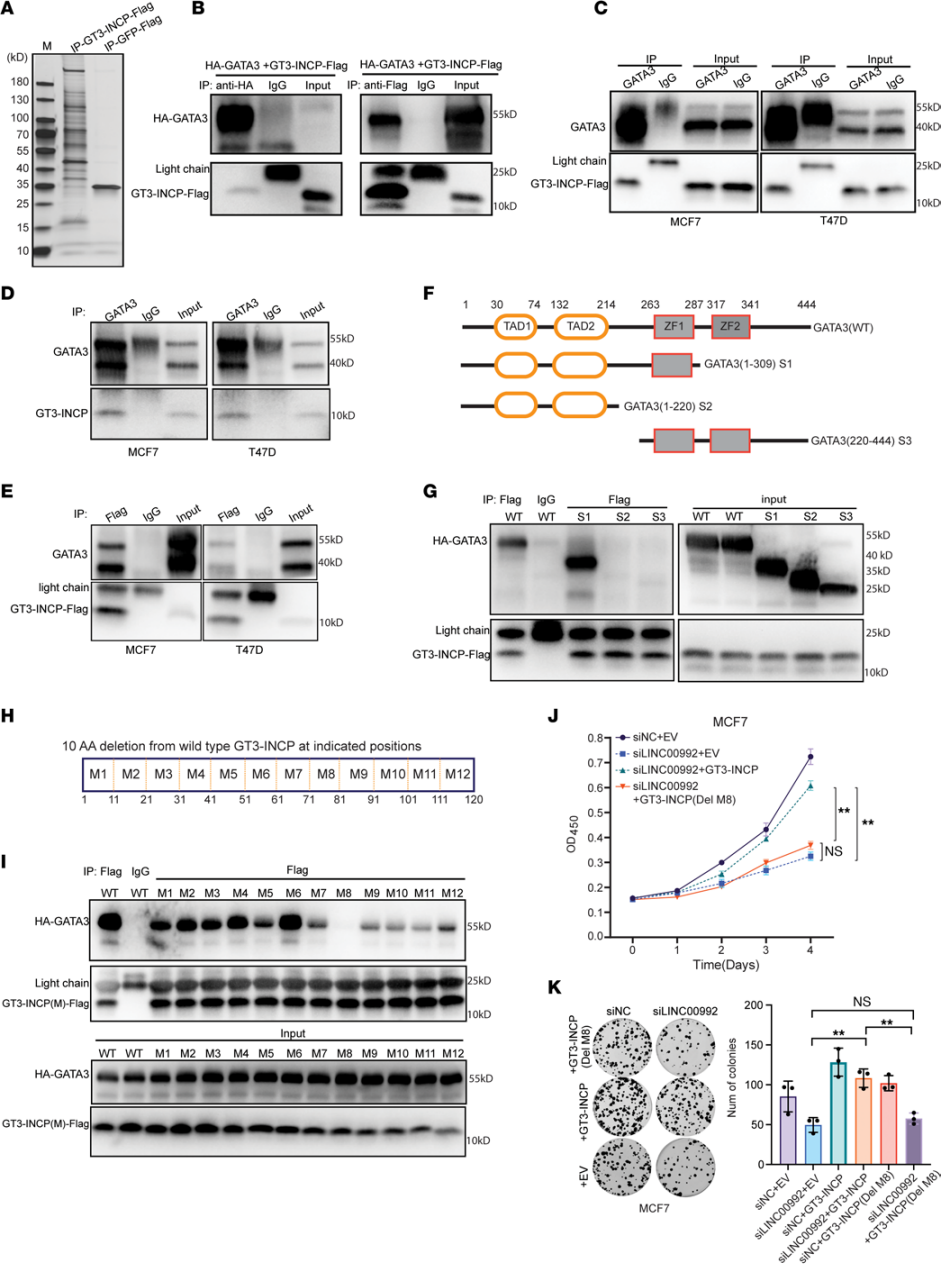
GT3-INCP interacts with GATA3. (**A**) Silver staining showing the proteins enriched by co-IP of FLAG-tagged GT3-INCP (IP-GT3-INCP-Flag) compared with the negative control FLAG-tagged GFP (IP-GFP-Flag) in MCF7 cells. Whole-cell lysates of (**B**) HEK293FT cells transfected with HA-tagged GATA3 (HA-GATA3) and FLAG-tagged GT3-INCP (GT3-INCP-Flag), (**C**) MCF7 and T47D cells stably expressing GT3-INCP-Flag, or the chromatin-bound extracts of (**D**) MCF7/T47D cells or (**E**) cells stably expressing GT3-INCP-Flag were immunoprecipitated with the indicated antibodies, followed by immunoblot analysis. Rabbit or mouse IgG was used as a negative control. (**F**) Diagram illustrating different domains of the full-length GATA3 and 3 truncation mutants (S1–S3). (**G**) Lysates of HEK293FT cells cotransfected with HA-tagged wild-type or mutant GATA3 and GT3-INCP-Flag were immunoprecipitated with an anti-FLAG antibody or IgG and then analyzed by immunoblotting. (**H**) Diagram illustrating the deletion mutants generated from the full-length GT3-INCP (M1–M12). (**I**) Lysates of HEK293FT cells cotransfected with HA-tagged GATA3 and FLAG-tagged wild-type or mutant GT3-INCP were immunoprecipitated with an anti-FLAG antibody or IgG and then analyzed by immunoblotting. (**J**) MCF7 cells stably transduced with the empty vector control (EV) or the indicated ORFs were transfected with siNC or a LINC00992-targeting siRNA. Cell growth was monitored by CCK-8 assay. (**K**) MCF7 cells stably transduced with EV or the indicated ORFs were transfected with siNC or a LINC00992-targeting siRNA, and were then assessed for colony formation. Representative pictures of clonogenic growth and a bar graph quantifying the colonies formed by these cells are shown. Data in **A**–**E**, **G**, and **I** are representative of 3 independent experiments. Data in **J** and **K** are shown as mean ± SD (*n* = 3). **P < 0.01 by 1-way ANOVA with Tukey’s multiple-comparison test. NS, not significant (*P* > 0.05).

To understand the molecular mechanism underlying the tumor-promoting function of GT3-INCP and systematically identify its interacting proteins, we performed affinity purification using an anti-FLAG antibody followed by MS (AP-MS) in MCF7 cells that stably expressed FLAG-tagged GT3-INCP or FLAG-tagged GFP in which the FLAG-tagged GFP served as a negative control (see Methods). Silver staining showed an enrichment of clear and specific bands from the co-IP of FLAG-tagged GT3-INCP in comparison with that of FLAG-tagged GFP (Figure 5A), suggesting a good quality of the AP experiment. We identified 37 proteins that were uniquely detected in the AP-MS for GT3-INCP, but not in that of GFP negative control (Methods and Supplemental Table 3), and showed a significantly elevated RNA expression in luminal BC tumors compared with the normal breast tissue (log_2_[fold change] ≥ 1, FDR < 0.01), representing candidates that may exert a tumor-promoting function together with GT3-INCP. Among the genes showing the top-ranked fold changes in expression between luminal tumor and normal breast tissue, we found an interesting candidate, GATA3, which is a member of the GATA family of transcription factors that is essential to the establishment and maintenance of luminal epithelial cell identity during mammary gland development (30) as well as a master regulator of T cell and innate lymphoid cell development (31). GATA3 is one of the most frequently mutated genes in BC (27). Its expression is highly correlated with that of ER and is a prominent marker of ER^+^ primary luminal BC tumors (27, 32). Consistent with its specific expression in luminal BC, GATA3 was identified as a highly selective dependency of luminal BC, along with ER and FOXA1, from systematic genome-wide RNAi- (33) or CRISPR-based (34) screens across hundreds of cancer cell lines. GATA3 has also been shown to be part of the core transcriptional regulatory circuitry in MYCN-amplified neuroblastoma cells (35) and plays an oncogenic role in high-grade serous ovarian carcinoma (36). Furthermore, GATA3 is required for estrogen-stimulated proliferation of ER^+^ BC cells (37). It has also been shown to be located at a large fraction of ER binding sites (38, 39) on chromatin and its consensus motif is enriched around ER binding sites (39, 40) in ER^+^ BC cells. It has been proposed that GATA3 may regulate ER-chromatin binding, at least partially by modulating enhancer accessibility (38, 41). GATA3, ER, and FOXA1 form a master cell-type-specific transcriptional regulatory network (37–39) that governs the phenotypes of hormone-dependent luminal BC. The reciprocal co-IP of HA-tagged GATA3 and FLAG-tagged GT3-INCP in HEK293FT cells (Figure 5B) and GATA3 and FLAG-tagged GT3-INCP in MCF7 and T47D cells confirmed their interaction (Figure 5C and Supplemental Figure 4A). Co-IP of the chromatin fraction also confirmed the interaction between GT3-INCP and GATA3 on chromatin (Figure 5, D and E, and Supplemental Methods). Importantly, siRNA-mediated LINC00992 depletion did not affect GATA3 protein levels (Supplemental Figure 4B), indicating that GT3-INCP interacted with GATA3, but did not regulate its expression.

Human GATA3 protein contains 2 transactivation domains (TAD1 and TAD2) and 2 highly conserved zinc-finger domains (ZF1 and ZF2) that are shared within the GATA family (42) (Figure 5F). Both TAD1 and TAD2 are required for GATA3 activity in reporter assays (42). The ZF2 domain of GATA3 is necessary and sufficient for binding to the GATA3 consensus recognition sequence in vitro, whereas deletion of the ZF1 domain has no impact on GATA3 binding to the same consensus sequence in vitro (42). To determine the regions in GATA3 that are important for mediating its binding to GT3-INCP, we generated a series of GATA3 truncation mutants (S1, aa 1–309; S2, aa 1–220; S3, aa 220–444) based on its domain architecture and performed co-IP of individual HA-tagged truncation mutants with FLAG-tagged GT3-INCP (Figure 5G) in HEK293FT cells. We found that deletion of the ZF2 domain did not affect the interaction with GT3-INCP, indicating that the ZF2 domain was not required for the GT3-INCP–GATA3 interaction. In addition, both the region (aa 1–220) that contains the TAD1 and TAD2 domains and the region (aa 221–309) that contains the ZF1 domain were involved in the interaction with GT3-INCP, but neither alone was sufficient for mediating this interaction (Figure 5G). To gain insight into the structural basis of the interaction between GT3-INCP and GATA3, we computationally predicted the 3-dimensional (3D) structure of full-length GATA3 (Supplemental Methods) using 2 state-of-the-art methods, AlphaFold (43, 44) and I-TASSER-MTD (45, 46). Although the GATA3 3D structures predicted by AlphaFold and I-TASSER-MTD were quite different, they shared the common feature that most parts of the GATA3 protein (except for the ZF1 and ZF2 domains) were not well structured, including the TAD1 and TAD2 domains (Supplemental Figure 4C). The computational prediction that the ZF1 and ZF2 domains adopted a well-defined structure was consistent with their experimentally determined structures (Supplemental Figure 4D). These results suggest that GATA3 alone may be largely unstructured and may undergo a conformational change when it interacts with GT3-INCP.

To map the regions in GT3-INCP that are required for the binding of GT3-INCP to GATA3, we constructed a series of deletion mutants with a removal of every 10-aa fragment (M1–M12) along the full-length GT3-INCP (Figure 5H). We found that only deletion of the M8 fragment (Del-M8; aa 71–80) completely abolished the binding of GT3-INCP to GATA3 (Figure 5I), indicating that this region is essential to GT3-INCP–GATA3 interactions. Interestingly, the 3D structures of GT3-INCP predicted by AlphaFold2 (43, 47) and I-TASSER (48, 49) (Supplemental Methods) both suggest that GT3-INCP may adopt a helix bundle structure and M8 may be in the loop region (Supplemental Figure 4, E and F).

To determine the role of the GT3-INCP–GATA3 interaction in mediating the tumor-promoting function of GT3-INCP, we performed rescue experiments to investigate whether the inhibition of ER^+^ luminal BC cell growth and colony formation caused by siRNA-mediated LINC00992 depletion can be reversed by overexpressing wild-type GT3-INCP or the Del-M8 mutant that showed a defective interaction with GATA3. We found that overexpression (Supplemental Figure 4, G and H) of the wild-type GT3-INCP, but not the Del-M8 mutant, rescued the loss-of-function effects of LINC00992 on cell growth (Figure 5J and Supplemental Figure 4I) and colony formation (Figure 5K and Supplemental Figure 4J). Collectively, our data indicate that the interaction between GT3-INCP and GATA3 is important for mediating the tumor-promoting function of GT3-INCP.

### GT3-INCP and GATA3 coregulate a common expression program impacting the genes associated with estrogen response/cell proliferation.

**Figure 6:**
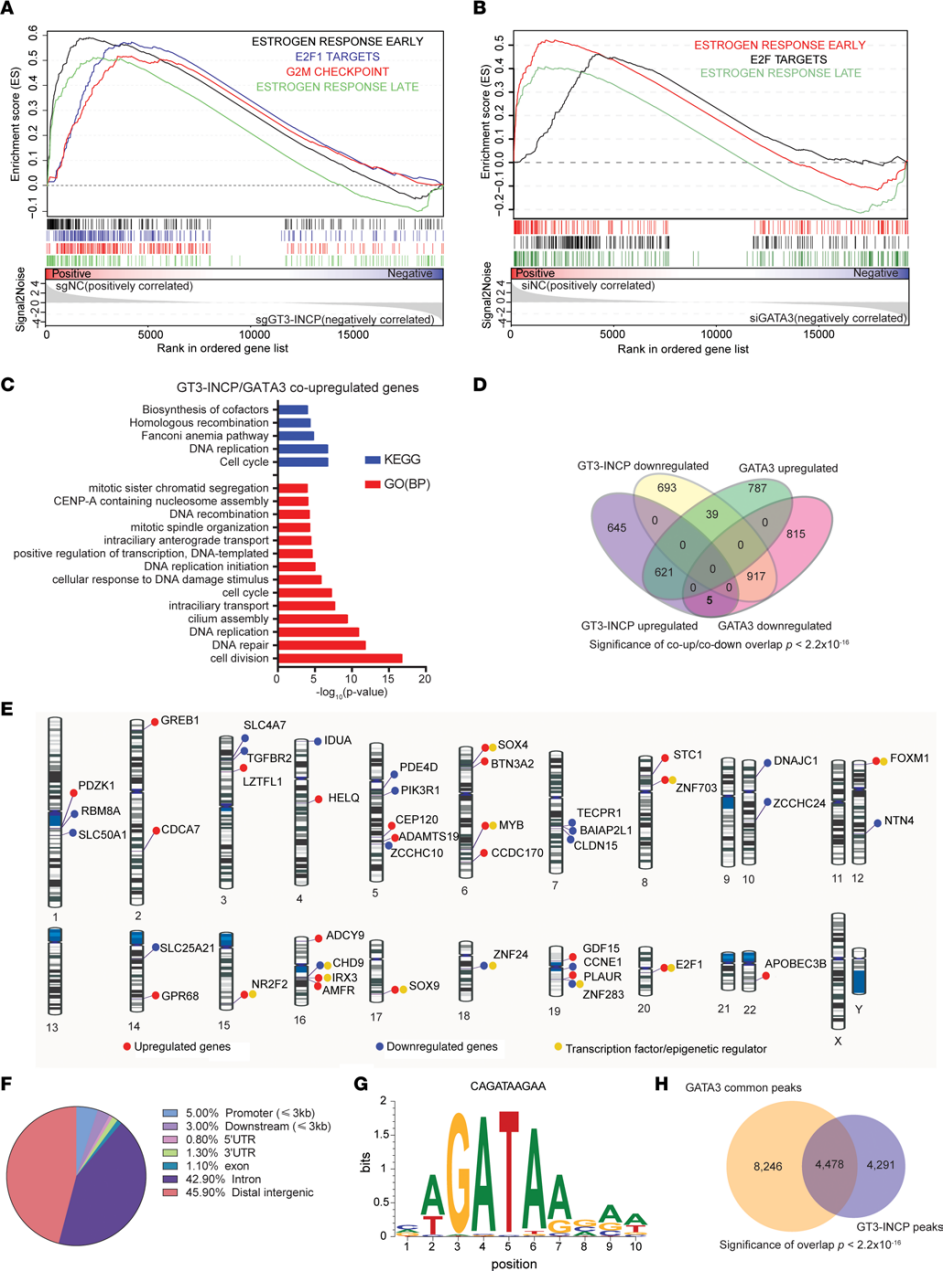
GT3-INCP and GATA3 coregulate a common gene expression program. Gene set enrichment analysis (GSEA) with the Hallmark gene sets showing the top enriched gene sets downregulated following (**A**) GT3-INCP knockout or (**B**) GATA3 knockdown. (**C**) Bar plot showing the top enriched Gene Ontology biological process (BP) terms and KEGG pathways ranked by –log_10_(*P* value), based on the functional enrichment analysis of protein-coding genes co-upregulated by GT3-INCP and GATA3. (**D**) Venn diagram showing the overlap between the genes downregulated and upregulated by GT3-INCP and GATA3. (**E**) Ideogram showing the chromosomal location/cytoband of the BC risk genes that are co-upregulated (red) or co-downregulated (blue) by GT3-INCP and GATA3. Those that are transcriptional factors/epigenetic regulators are shown in yellow. (**F**) The genome-wide distribution of GT3-INCP binding sites identified from ChIP-seq data in MCF7 cells. (**G**) The sequence logo of the top motif (human GATA3 motif) identified by motif enrichment analysis (Supplemental Methods) from the GT3-INCP binding sites. (**H**) Venn diagram showing the overlap between the GT3-INCP binding sites and high-confidence common GATA3 binding sites that were shared among 3 GATA3 ChIP-seq data sets (GSE32465 and GSE128460) in MCF7 and T47D cells. Fisher’s exact test was used to assess the statistical significance of the Venn diagram overlap (**D** and **H**).

With the finding that GT3-INCP and GATA3 interacted with each other, we hypothesized that GT3-INCP and GATA3 may coregulate common functionally important downstream targets. To test this hypothesis, we first performed RNA-seq to determine the changes in gene expression upon sgRNA-mediated GT3-INCP knockout or siRNA-mediated GATA3 knockdown in MCF7 cells (Supplemental Tables 4 and 5). Gene set enrichment analyses (GSEAs) (50) that used the Hallmark gene sets from the Molecular Signatures Database (51) revealed a significant downregulation of estrogen response genes and E2F1 targets in the sgRNA-mediated knockout group for GT3-INCP (Figure 6A and Supplemental Figure 5A) and siRNA-mediated knockdown group for GATA3 (Figure 6B), suggesting their role in regulating expression of the genes related to estrogen response and E2F1 activity. In particular, 112 estrogen response genes annotated by Molecular Signature Database Hallmark gene sets (51) were downregulated by sgRNA-mediated GT3-INCP knockout (Supplemental Figure 5, B and C). To gain insight into the important pathways that may be coregulated by GT3-INCP/GATA3, we performed Gene Ontology (GO) analysis of the common downstream targets, the expression of which was coregulated by GT3-INCP knockout and GATA3 knockdown (Supplemental Methods). We found that the protein-coding genes co-upregulated by GT3-INCP and GATA3 (i.e., co-downregulated by their depletion; Supplemental Table 6) showed an enrichment of pathways/biological processes linked to DNA replication, cell cycle/division, and DNA repair (Figure 6C). In contrast, the genes co-downregulated by GT3-INCP and GATA3 (Supplemental Table 6) showed an enrichment of pathways/biological processes such as antigen presentation, autophagy, protein transport, etc. (Supplemental Figure 5D). There were 1,649 upregulated and 1,271 downregulated protein-coding genes (|log_2_[fold change]| ≥ log_2_[1.5] and FDR < 0.05) upon sgRNA-mediated GT3-INCP knockout (Figure 6D). The GATA3 knockdown resulted in 1,737 upregulated and 1,447 downregulated protein-coding genes (Figure 6D). Consistent with our hypothesis that GT3-INCP and GATA3 coregulate common target gene expression, a statistically significant (Fisher’s exact test, *P* < 2.2 × 10^–16^) number of upregulated (917) and downregulated (621) protein-coding genes were shared following GT3-INCP knockout and GATA3 knockdown.

Consistent with the GT3-INCP knockout result, there was a significant downregulation of estrogen response genes and E2F1 targets upon siRNA-mediated LINC00992 knockdown (Supplemental Figure 5E). The protein-coding genes co-downregulated by knockdown of LINC00992 and GATA3 also showed an enrichment of pathways/biological processes linked to DNA replication, cell cycle/division, and DNA repair (Supplemental Figure 5F). In addition, the protein-coding genes that were up-/downregulated by sgRNA-mediated GT3-INCP knockout largely overlapped (Fisher’s exact test, *P* < 2.2 × 10^–16^) with the ones regulated by LINC00992 knockdown (Supplemental Figure 5G). For the downstream analyses, we focused on the common targets that showed consistent up-/downregulation upon sgRNA-mediated GT3-INCP knockout and siRNA-mediated LINC00992 knockdown. Importantly, many of the common targets coregulated by GT3-INCP and GATA3 are BC susceptibility/risk genes based on existing literature (Supplemental Methods), more than 25% of which are transcription factors or epigenetic regulators (Figure 6E and Supplemental Table 7).

To identify the direct targets of GT3-INCP transcriptional regulation, we performed chromatin IP followed by next-generation sequencing (ChIP-seq) using an anti-FLAG antibody in the MCF7 cells that stably expressed FLAG-tagged GT3-INCP (Supplemental Methods) to define the genome-wide GT3-INCP binding sites. We identified a total of 8,937 GT3-INCP binding sites (FDR < 0.01; Supplemental Table 8) that were predominantly at distal intergenic (>3 kb from transcription start or termination sites) and intronic regions (Figure 6F). The conservation plot showed that the sequences from the GT3-INCP binding sites were more conserved than their flanking regions (Supplemental Figure 5H). Interestingly, we found that the top-ranked motifs enriched in the GT3-INCP binding sites were GATA family member motifs (Supplemental Methods and Supplemental Table 8), with the top motif being the human GATA3 motif (Figure 6G). Consistent with the motif analysis result, more than 50% of the GT3-INCP binding sites (Fisher’s exact test, *P* < 2.2 × 10^–16^; Figure 6H) overlapped with the high-confidence common GATA3 binding sites that were shared between different GATA3 ChIP-seq data sets (Supplemental Figure 5I), further supporting the model in which GT3-INCP interacts with GATA3 on chromatin to coregulate a common gene expression program.

### GT3-INCP and GATA3 bind to common cis regulatory elements and upregulate the expression of MYB and PDZK1.

**Figure 7:**
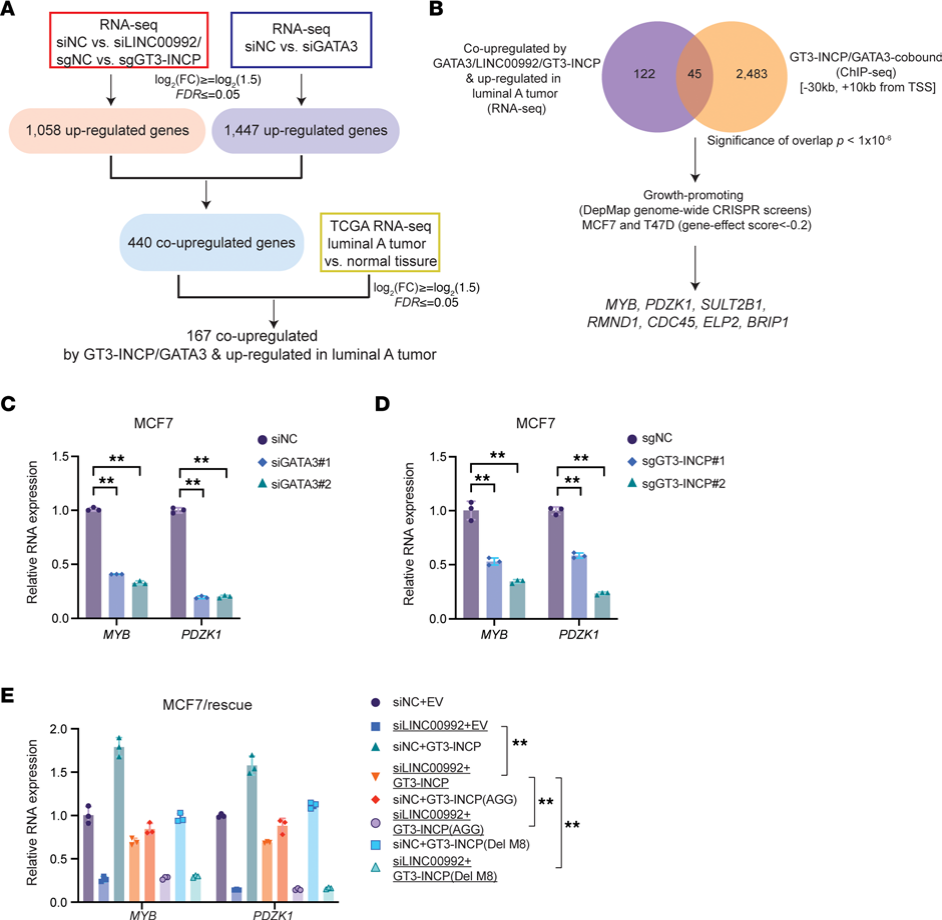
GT3-INCP and GATA3 upregulate MYB and PDZK1 expression. (**A**) Workflow for identifying the protein-coding genes co-upregulated by GT3-INCP and GATA3 and upregulated in luminal A BC compared with normal breast tissue. (**B**) Workflow for identifying the key targets that were potentially important for mediating the tumor-promoting function of the GT3-INCP/GATA3 axis in ER^+^ luminal BC. Venn diagram showing the overlap between the protein-coding genes that were co-upregulated by GT3-INCP/GATA3 and upregulated in luminal BC tumors, and the genes that harbored common GT3-INCP/GATA3 binding site(s). qRT-PCR analysis showing *MYB* and *PDZK1* expression changes in MCF7 cells following (**C**) GATA3 knockdown or (**D**) GT3-INCP knockout. (**E**) Upon LINC00992 knockdown, the rescue effect of ectopic expression of the wild-type or mutant GT3-INCP (Del-M8 or AGG mutation in start codon), with respect to the empty vector control (EV), on *MYB* and *PDZK1* mRNA expression was assessed by qRT-PCR in MCF7 cells. Fisher’s exact test was used to assess the statistical significance of the Venn diagram overlap (**B**). Data in **C**–**E** are shown as mean ± SD (*n* = 3). **P < 0.01 by 1-way ANOVA with Dunnett’s multiple-comparison test. NS, not significant (*P* > 0.05).

To identify the common direct targets of GT3-INCP and GATA3 that are important for mediating their tumor-promoting function, we performed an integrated analysis using the RNA-seq and ChIP-seq data in ER^+^ BC cells, together with TCGA data (Figure 7, A and B). We identified 45 protein-coding genes (Supplemental Table 9) that were co-upregulated by GT3-INCP and GATA3 and significantly upregulated in luminal A BC compared with normal breast tissues, and harbored at least 1 common GT3-INCP/GATA3 binding site within a 40-kb window (–30 kb to +10 kb) around their transcription start sites (Fisher’s exact test, *P* < 1 × 10^–6^; Figure 7B). These genes were common direct targets of GT3-INCP and GATA3 and the candidates that may exert a tumor-promoting function. Furthermore, we integrated the cancer dependency map (DepMap) data (https://depmap.org/portal/) (34) that was generated from genome-wide CRISPR-based screens across hundreds of cancer cell lines to identify 7 out of the 45 genes that showed a consistent growth-promoting phenotype (gene-effect score < –0.2) in both MCF7 and T47D cells (Figure 7B and Supplemental Table 9).

Among these 7 genes, *MYB* and *PDZK1* are the only 2 BC susceptibility/risk genes (Supplemental Table 7). MYB is a transcription factor and a key regulator of stem/progenitor cells in the bone marrow and colonic crypts (52). It plays an important role in leukemogenesis and is overexpressed in solid tumors such as colorectal cancer and BC (52). MYB overexpression is strongly associated with ER^+^ BC (27) and MYB is associated with BC susceptibility/risk (53). MYB is a direct target of ER and is required for ER^+^ BC cell proliferation in vitro (54) and tumor growth in vivo (55). PDZK1 is an adaptor protein that contains 4 PDZ-interacting domains. It is critical for maintaining levels of the scavenger receptor class B, type I, the receptor of high-density lipoprotein (HDL) that controls HDL metabolism (56). It is overexpressed in ER^+^ BC compared with ER^–^ BC (57) and is associated with BC susceptibility (58). *PDZK1* is upregulated upon estrogen stimulation (57, 59) and promotes estrogen-mediated growth of ER^+^ BC cells (59).

Consistent with our RNA-seq data, qRT-PCR analysis confirmed that siRNA-mediated knockdown of GATA3 (Supplemental Figure 6, A and B) or LINC00992, or sgRNA-mediated knockout of GT3-INCP, reduced the RNA expression of MYB and PDZK1 (Figure 7, C and D, and Supplemental Figure 6, C–F). To further confirm the GT3-INCP protein function and the role of the GT3-INCP–GATA3 interaction in regulating the RNA expression of MYB and PDZK1, we performed rescue experiments to investigate whether the targeted downregulation by siRNA-mediated LINC00992 silencing can be reversed by overexpressing wild-type GT3-INCP, Del-M8 GT3-INCP, or GT3-INCP with the AGG start codon mutation. It is noted that overexpression of wild-type GT3-INCP, but not the mutant ones, reversed the downregulation of MYB and PDZK1 caused by LINC00992 knockdown in MCF7 and T47D cells (Figure 7E and Supplemental Figure 6G). These results indicate that GT3-INCP protein and the GT3-INCP–GATA3 interaction are important for regulating MYB and PDZK1 expression.

**Figure 8:**
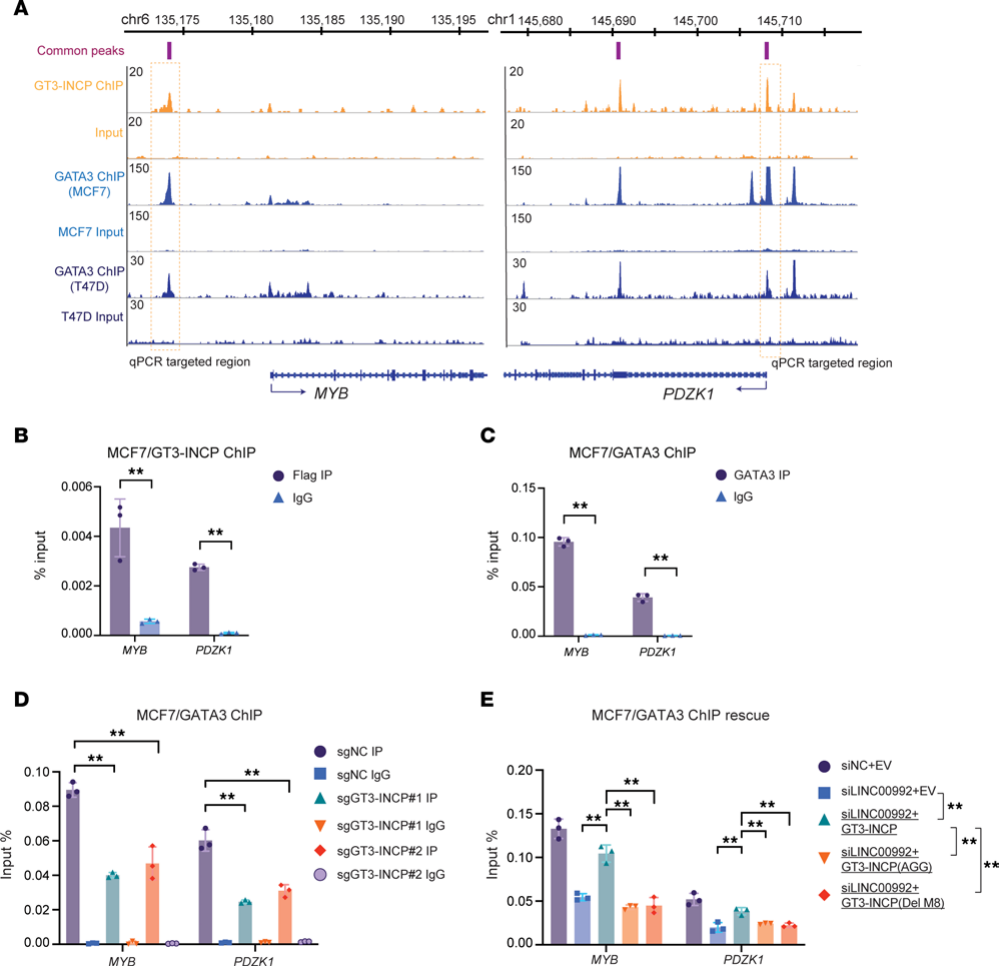
GT3-INCP facilitates the binding of GATA3 to the common *cis* regulatory elements of *MYB* and *PDZK1*. (**A**) The GT3-INCP and GATA3 ChIP-seq signal and peaks around *MYB* and *PDZK1* in MCF7 and T47D cells. ChIP-qPCR validation of (**B**) GT3-INCP and (**C**) GATA3 binding to the ChIP-seq peaks around *MYB* and *PDZK1* with the indicated antibodies in MCF7 cells stably expressing the FLAG-tagged GT3-INCP. (**D**) ChIP-qPCR analysis for assessing the effect of GT3-INCP knockout on GATA3 occupancy on its binding sites around *MYB* and *PDZK1* in MCF7 cells. (**E**) Upon LINC00992 knockdown, ChIP-qPCR analysis was performed to assess the rescue effect of ectopic expression of wild-type GT3-INCP or mutant GT3-INCP (Del-M8 or AGG), with respect to the EV control, on the GATA3 occupancy on its binding sites in MCF7 cells. Data in **B**–**E** are shown as mean ± SD (*n* = 3). **P < 0.01 by 2-tailed, unpaired Student’s *t* test (**B** and **C**) or 1-way ANOVA with Dunnett’s multiple-comparison test (**D** and **E**). NS, not significant (*P* > 0.05).

Further ChIP-qPCR analyses revealed the binding of GT3-INCP and GATA3 to the ChIP-seq–identified *cis* regulatory elements (Figure 8A) of *MYB* and *PDZK1* (Figure 8, B and C, and Supplemental Figure 6, H and I), supporting the notion that *MYB* and *PDZK1* are common direct targets of GT3-INCP and GATA3.

Based on our findings that GT3-INCP interacted with GATA3 on chromatin and did not regulate GATA3 protein level (Supplemental Figure 4B), we hypothesized that GT3-INCP may regulate the expression of *MYB* and *PDZK1* by facilitating GATA3 binding to their *cis* regulatory elements. To test this hypothesis, we evaluated the effect of GT3-INCP knockout on GATA3 binding to the *cis* regulatory elements of *MYB* and *PDZK1* by ChIP-qPCR. Indeed, GATA3 occupancy on these binding sites was significantly reduced upon GT3-INCP knockout (Figure 8D and Supplemental Figure 6J). To further confirm the GT3-INCP protein function and the role of GT3-INCP–GATA3 interactions in regulating GATA3 binding to the common *cis* regulatory elements, we investigated whether reduction of GATA3 binding to the *cis* regulatory elements by siRNA-mediated LINC00992 silencing can be reversed by overexpressing wild-type GT3-INCP or the mutant GT3-INCP (Del-M8 or AGG). We found that overexpression of wild-type GT3-INCP, but not the mutant ones, largely reversed the reduction in GATA3 binding to the *cis* regulatory elements of *MYB* and *PDZK1* that was caused by LINC00992 knockdown (Figure 8E and Supplemental Figure 6K). Collectively, these results indicated that GT3-INCP protein and GT3-INCP–GATA3 interactions are important for facilitating GATA3 binding to the common *cis* regulatory elements.

### GT3-INCP is upregulated by estrogen/ER and is important for estrogen-dependent cell growth and estrogen-regulated gene expression.

**Figure 9:**
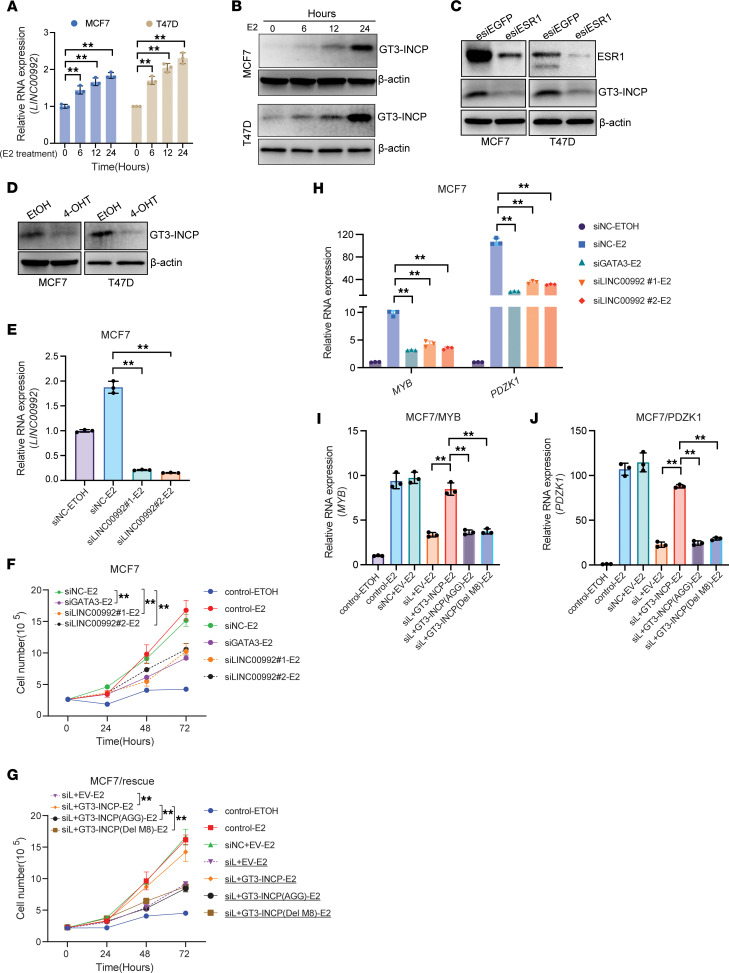
GT3-INCP is upregulated by estrogen/ER and is important for estrogen-dependent cell growth/gene expression. (**A**) qRT-PCR analysis of *LINC00992* RNA expression and (**B**) Western blot analysis of GT3-INCP expression in MCF7 and T47D cells upon β-estradiol (E2) treatment (30 nM) for the indicated time intervals. Western blot analysis of GT3-INCP expression (**C**) in MCF7/T47D cells transfected with ESR1-targeting endoribonuclease-prepared siRNA (esiRNA) or GFP-targeting esiRNA EGFP (esiEGFP), or (**D**) in the cells treated with 15 μM 4-hydroxytamoxifen (4-OHT; Sigma-Aldrich, SML1666) or vehicle (ethanol, ETOH) control. (**E**) qRT-PCR analysis of *LINC00992* RNA expression in MCF7 cells that were transfected with the negative control siRNA (siNC) or LINC00992-targeting siRNAs (siLINC00992), after E2 (30 nM) or ETOH vehicle treatment. (**F**) After E2/ETOH treatment, the numbers of MCF7 cells treated with transfection reagent (control) or transfected with siNC or GATA3-targeting siRNAs (siGATA3) or siLINC00992 were counted every 24 hours for 72 hours. (**G**) After E2/ETOH treatment, the number of MCF7 cells that were treated with transfection reagent (control) or the MCF7 cells that were transduced with the empty vector (EV) or the indicated ORFs and transfected with siNC/siLINC00992 (siL) was monitored for 72 hours. (**H**) qRT-PCR analysis of *MYB* and *PDZK1* RNA expression in MCF7 cells that were transfected with siNC, siGATA3, or siLINC00992, after E2/ETOH treatment. qRT-PCR analysis of (**I**) *MYB* and (**J**) *PDZK1* RNA expression in the MCF7 cells that were treated with transfection reagent (control) or the MCF7 cells that were transduced with EV or the indicated ORFs and transfected with siNC/LINC00992-targeting siRNA (siL), after E2/ETOH treatment. Data in **A** and **E**–**J** are shown as mean ± SD (*n* = 3). **P < 0.01 by 1-way ANOVA with Dunnett’s multiple-comparison test. NS, not significant (*P* > 0.05). Data in **B**–**D** are representative of 3 independent experiments.

Given our finding that GT3-INCP was upregulated in ER^+^ BC tumors, we sought to determine whether GT3-INCP expression was regulated by estrogen and ER. We found that between 6 and 24 hours after estrogen/β-estradiol (E2) treatment (30 nM) of the ER^+^ BC cells that were cultured in E2-deprived condition (see Methods), the expression of GT3-INCP showed an increase at both the RNA and protein level (Figure 9, A and B). In addition, either siRNA-mediated ER knockdown or pharmacological inhibition of ER with the antagonist 4-hydroxytamoxifen (4-OHT) reduced GT3-INCP expression (Figure 9, C and D). These results indicate that GT3-INCP expression is upregulated by estrogen and ER. Because GT3-INCP regulated the expression of many E2 responsive genes (Supplemental Figure 5, B and C), we further investigated the role of GT3-INCP in E2-dependent growth of ER^+^ BC cells. We found that siRNA-mediated knockdown of GATA3 (Supplemental Figure 7A) or LINC00992 (Figure 9E) inhibited E2-stimulated cell growth (Figure 9F and Supplemental Figure 7B). Overexpression of wild-type GT3-INCP, but not the mutant ones (Del-M8 or AGG), largely rescued the E2-dependent cell growth defect caused by LINC00992 knockdown (Figure 9G and Supplemental Figure 7C). Moreover, GATA3 or LINC00992 knockdown reduced estrogen-regulated expression of MYB and PDZK1 (Figure 9H and Supplemental Figure 7D). Overexpression of wild-type GT3-INCP, but not the mutant ones (Del-M8 or AGG), largely reversed the reduction in the E2-stimulated MYB (Figure 9I and Supplemental Figure 7E) and PDZK1 (Figure 9J and Supplemental Figure 7F) expression caused by LINC00992 knockdown. Taken together, these results indicate that GT3-INCP protein and GT3-INCP–GATA3 interactions are important for E2-dependent cell growth and E2-regulated expression of MYB and PDZK1.

## Discussion

lncRNAs are an emerging class of regulators of gene expression that play critical roles in diverse biological processes, including cell fate decision, immune response, and cellular stress response. Like protein-coding genes, lncRNAs can exert tumor-promoting/suppressing functions and may serve as independent diagnostic or prognostic biomarkers. Increasing evidence supports the notion that some of the lncRNAs encode functional proteins that play important developmental and physiological roles in different metazoan species. Aside from lncRNAs, recent studies (60, 61) in human cells revealed that cryptic translation of noncanonical ORFs within other annotated noncoding regions such as UTRs can produce functional proteins. Different from the traditional view of 5′-UTR–encoded ORFs (upstream ORFs [uORFs]) as *cis*-acting translational control elements, multiple uORF-encoded microproteins have been found to form stable complexes with the main protein encoded on the same mRNA (60). In the current study, we integrated ribo-seq and a CRISPR/Cas9 knockout pooled screen (23) with large-scale computational analysis of TCGA data, and identified the ER^+^ BC dependency on 28 functional cryptic ORFs encoded by lncRNAs, the expression of which was upregulated in luminal BC compared with normal breast tissues. Among the identified cryptic-ORF dependencies, we validated in vitro and/or in vivo tumor-promoting functions for 2 of them that are encoded by *LINC00992* and *GATA3-AS1*. Interestingly, the overexpression of LINC00992 and GATA3-AS1 in tumors or cancer cell lines was previously associated with chemoresistance (62, 63). These findings suggest that although our CRISPR screen was performed in the absence of drugs, some of the identified functional cryptic ORFs might play an important role in therapeutic resistance of ER^+^ BC, which warrants further investigation.

Cryptic lncRNA-encoded proteins have been shown to perform functions in different subcellular compartments such as the sarcoplasmic reticulum membrane, cytosol, and mitochondria. However, their role in transcriptional regulation in the nucleus is largely unknown. Our findings that GT3-INCP interacted with GATA3, a GATA family transcription factor that is key to mammary gland development and an essential lineage-specific dependency of ER^+^ luminal BC, to coregulate the expression of BC susceptibility genes and/or the genes key to the growth/proliferation of ER^+^ BC cells, demonstrate a lncRNA-encoded protein as an integrated component of a master transcriptional regulatory network that drives the aberrant transcription in cancer, underscoring the underappreciated and important role of lncRNA-encoded proteins in transcriptional regulation. Interestingly, we found that the ZF2 domain that is necessary and sufficient for sequence-specific DNA binding of GATA3 is not required for GT3-INCP–GATA3 interactions, suggesting that GT3-INCP might modulate GATA3 transactivation activity rather than its DNA binding activity to coregulate the expression GATA3 target genes.

Human lncRNAs generally show a highly context-specific expression and function. The current study focuses on the cryptic proteins encoded by lncRNAs in ER^+^ BC. Therefore, we anticipate that our study only revealed a small fraction of the functional human proteins encoded by lncRNAs. Our integrative genomic approach is generally applicable to other biological contexts and promises to open new avenues for identifying cryptic functional proteins encoded by lncRNAs in complex diseases other than ER^+^ BC. The past efforts of cancer therapeutic target/diagnostic biomarker discovery have been predominantly focused on the annotated human proteome. Our findings indicate that the cryptic proteome encoded by lncRNAs represents an understudied proteome, part of which is hijacked by cancer cells to promote their fitness, and may be a new and untapped space for therapeutic/diagnostic target discovery.

## Methods

### Cell lines, plasmids, and antibodies.

Human BC cell lines MCF7, T47D, ZR75-1, MDA-MB-231, and human breast epithelial cell line MCF10A were obtained from American Type Culture Collection (ATCC) and cultured according to ATCC’s instructions. Human embryonic kidney cell line HEK293FT were obtained from the Characterized Cell Line Core facility at MD Anderson Cancer Center (MDACC) and cultured in Dulbecco’s modified Eagle’s medium (DMEM; Hyclone, SH30022.01). MCF7, T47D, and ZR75-1 cells were cultured in RPMI-1640 (Hyclone, SH30027.1). MCF10A cells were cultured in DMEM/F12 (Invitrogen, 11330-032) with 5% horse serum (Invitrogen, 16050-122), 20 ng/mL EGF (STEMCELL, 78006.1), 0.5 mg/mL hydrocortisone (STEMCELL, 74142), and 10 mg/mL insulin (Sigma-Aldrich, I3536). All culture media were supplemented with 10% FBS (Gibco, 10437-028) and 1% penicillin/streptomycin (Corning, 30-002-CI). All cell lines were cultured in an incubator (Thermo Fisher Scientific, HEARCELL VIOS 160i) with 5% CO_2_ at 37°C. GATA3 (catalog 116747) and pLenti-CMV-Blast DEST (w118-1) (catalog 17452) expression plasmids were obtained from Addgene. The DNA sequence of 5′-UTR-ORF-LINC00992 or ORF-LINC00992 was synthesized by Twist Bioscience and subcloned into the pLenti-CMV-Blast DEST vector. The wild-type and mutant GT3-INCP were subcloned into pLVX-puro with 3×FLAG tag. The wild-type and GATA3 mutants were subcloned into pcDNA3.1 with an HA tag. The sequences of all the plasmids were confirmed by Sanger sequencing. The antibodies used in this study include mouse anti-FLAG M2 Affinity Gel (Sigma-Aldrich, A2220), rabbit anti-GATA3 (CST, 5852), rabbit anti-HA-Tag (Proteintech, 51064-2-AP), mouse anti–β-actin monoclonal antibody (Proteintech, 66009-1-Ig), rabbit anti–β-tubulin polyclonal antibody (Proteintech, 10068-1-AP), rabbit anti-ER polyclonal antibody (Proteintech, 21244-1-AP), and custom rabbit anti–GT3-INCP (ABclonal) polyclonal antibody. Anti-GATA3 antibody [EPR16651] (Abcam, ab199428) was used for ChIP analysis.

### RNA isolation, cDNA synthesis, and quantitative PCR.

Cells were harvested 48 hours after siRNA transfection and total RNA was extracted using the RNeasy Mini kit (QIAGEN, 74104), according to the manufacturer’s instructions. cDNA synthesis was then performed with 1 μg of total RNA using the iScript cDNA Synthesis Kit (Bio-Rad, 1708890). qRT-PCR was performed using 2× Universal SYBR Green Fast qPCR Mix (ABclonal, RK21203) in the CFX96 Touch Real-Time PCR Detection System (Bio-Rad) according to the manufacturer’s instructions. All primers were synthesized by Sigma-Aldrich and their sequences are listed in Supplemental Table 10. Glyceraldehyde 3-phosphatedehydrogenase (*GAPDH*) was used as an internal control, and the fold change in gene expression level was calculated using the 2^–ΔΔCT^ method.

### LC-MS/MS analysis for detecting peptides derived from GT3-INCP.

The ectopically expressed FLAG-tagged and endogenously expressed GT3-INCP immunoprecipitated with an anti-FLAG/anti–GT3-INCP antibody were resolved in NuPAGE 10% Bis-Tris gels (Life Technologies) and the molecular weight region up to 25 kDa was excised and processed for in-gel digestion using trypsin. The tryptic peptides were analyzed on a nano-LC 1200 system (Thermo Fisher Scientific) coupled to an Orbitrap Fusion Lumos ETD (Thermo Fisher Scientific) mass spectrometer. The peptides were loaded on a 2-column setup using a precolumn trap of 2 cm × 100 μm size (Reprosil-Pur Basic C18 1.9 μm, Dr. Maisch GmbH) and a 20 cm × 75 μm analytical column (Reprosil-Pur Basic C18 1.9 μm, Dr. Maisch GmbH) with a 110-minute gradient of 6% to 30% acetonitrile/0.1% formic acid at a flow rate of 200 nL/min. The eluted peptides were directly electrosprayed into the mass spectrometer operated in data-dependent acquisition (DDA) mode or PRM mode. For DDA mode, the full MS scan was acquired in Orbitrap in the range of 300 to 1400 *m*/*z* at 120,000 resolution followed by MS2 in ion trap (HCD 32% collision energy) with 10-second dynamic exclusion time. For PRM mode, the target precursor ions corresponding to the new ORF peptide sequences were isolated in quadrupole with isolation width 1.6 *m*/*z* for the whole duration. The MS2 was carried out in ion trap (rapid scan, scan range 150–1800 *m*/*z*, automatic gain control 2 × 10^4^, max injection time 100 ms) using high-energy collisional dissociation (HCD) fragmentation (HCD 32% collision energy). The RAW MS files were processed with Proteome Discoverer 1.4 (Thermo Fisher Scientific) using Mascot 2.4 (Matrix Science) with percolator against the new protein sequence and the human protein NCBI RefSeq (https://www.ncbi.nlm.nih.gov/refseq/ Updated and accessed March 24, 2020.). The precursor ion tolerance and product ion tolerance were set to 20 ppm and 0.5 Da, respectively. Dynamic modification of oxidation on methionine, protein N-terminal acetylation, deamidation on N/Q, and carbamidomethyl on cysteine were allowed. The peptides identified from the Mascot results file were validated with 5% FDR and manually checked for correct assignment. The identification results and raw files were imported into Skyline software (MacCoss lab, University of Washington, Seattle, Washington, USA; https://skyline.ms/project/home/begin.view) for PRM analysis. The MS2 chromatograms were evaluated by selecting PRM in the acquisition method and using the ion trap as product mass analyzer with 0.5 *m*/*z* resolution.

### AP-MS–based mapping of protein-protein interactions.

MCF7 cells stably expressing FLAG-tagged GT3-INCP or FLAG-tagged GFP were lysed in Pierce IP Lysis Buffer (Thermo Fisher Scientific, 87787) with protease inhibitor and 10 mM PMSF (Thermo Fisher Scientific, 36978). The whole-cell lysates were incubated with anti-FLAG M2 agarose beads (Sigma-Aldrich, A2220) overnight with gentle rotation at 4°C. After incubation, the beads were washed 5 times with washing buffer (10 mM Tris [pH 7.4], 1 mM EDTA, 1 mM EGTA, pH 8.0, 150 mM NaCl, 1% Triton X-100) and resuspended in SDS-PAGE sample buffer (Bio-Rad, 1610747). The precipitated proteins on the beads were eluted by competition with 3×FLAG peptides (Sigma-Aldrich, F4799). The eluted proteins were resolved in SDS-PAGE and were sent to the Taplin MS facility (https://taplin.hms.harvard.edu/home) for LC-MS/MS analysis, as described previously (64). To identify the proteins that specifically interact with GT3-INCP, the following filters were applied: number of identified unique peptides ≥2 in the AP-MS of FLAG-tagged GT3-INCP and zero in that of FLAG-tagged GFP. Additional filters of differential expression between luminal A BC tumors and normal breast tissues (log_2_[fold change] ≥ 1, FDR < 0.01) were further applied to identify the candidate proteins (Supplemental Table 3) that may exert a tumor-promoting function in luminal BC.

### RNAi-mediated gene silencing, CRISPR/Cas9-mediated gene knockout, and ORF overexpression.

For the loss-of-function experiments using CRISPR/Cas9-mediated gene knockout in cell populations, the negative control sgRNA or gene-specific sgRNA was subcloned into the lentiCRISPR v2 (Addgene, 52961) vector. To produce lentiviruses, HEK293FT cells were cotransfected with pCMV-VSV-G, psPAX2, and sgRNA-expressing lentiCRISPR v2 plasmid using jetPRIME (Polyplus Transfection, 114-15). Lentiviruses were collected 48 hours after transfection and were then used to infect the cell lines in the presence of polybrene (Sigma-Aldrich, TR-1003) prior to puromycin selection for 4 days. The knockout efficiency of individual sgRNAs was determined by Western blotting after 10 days of puromycin selection, and then the cells were harvested for functional assays. For siRNA-mediated knockdown experiments, 1 negative control siRNA and 2 predesigned on-target siRNAs (Sigma-Aldrich) were used. A total of 1 × 10^5^ cells were plated in each well of 12-well plates. In each well, 40 pmol of siRNA was transfected into cells using Lipofectamine RNAiMAX Transfection Reagent (Thermo Fisher Scientific, 13778150), and total RNA was extracted 48 hours after transfection for qRT-PCR analysis of knockdown efficiency. For shRNA-mediated knockdown, the shRNA sequences were subcloned into the PLKO.1 TRC vector. To produce lentiviruses, HEK293FT cells were cotransfected with pCMV-VSV-G, psPAX2, and shRNA-expressing PLKO.1 TRC plasmid using jetPRIME. Lentiviruses were harvested 48 hours after transfection and then were used for infecting ER^+^ BC cells in the presence of polybrene prior to puromycin selection for 2 days. Total RNA and protein were harvested 4 days after puromycin selection. qRT-PCR and Western blotting were used to determine the shRNA-mediated knockdown efficiency at the RNA and protein level, respectively. 5′-UTR-GT3-INCP was amplified from the cDNAs extracted from MCF7 cells and subcloned into the pLenti-CMV-Blast DEST vector. The wild-type and GT3-INCP/ORF-GATA3-AS1 mutants were synthesized (Twist Bioscience) and subcloned into the pLVX-puro or pLenti-CMV-Blast DEST vector with FLAG tag. The wild-type and GATA3 mutants were subcloned into pcDNA3.1 with HA tag or pLenti-CMV-Blast DEST vector. For ORF overexpression in HEK293FT, the plasmids were transfected with jetPRIME transfection reagent. For the ORF overexpression in ER^+^ BC cells, lentivirus particles were produced in HEK293FT and then collected for transducing the cell lines. The expression was determined by Western blot assays and then collected for functional assays. All sgRNA, siRNA, and shRNA sequences are listed in Supplemental Table 10.

### Cell growth and gene expression analysis with estrogen treatment.

For estrogen treatment experiments, MCF7 and T47D cells were maintained in phenol red–free RPMI-1640 medium (Gibco, 11835-030) containing 5% charcoal-stripped FBS (Gibco, 12676-029) for 3 days, with medium changed each day, followed by E2 (Sigma-Aldrich, E2758) treatment (30 nM) for the indicated times. For the siRNA-based loss-of-function experiments, 8 × 10^4^ MCF7 or T47D cells or MCF7/T47D stably transduced with the empty vector (EV) control and the indicated ORFs, were seeded in 6-well plates in phenol red–free medium containing 5% charcoal-stripped FBS. After 24 hours, the cells were transfected with negative control siRNA (siNC) or the siRNAs targeting the indicated transcripts. The cells were maintained in phenol red–free medium containing 5% charcoal-stripped FBS for another 2 days, with medium changed each day. Then, the cells were treated with 30 nM E2 for the indicated times. The cell numbers were counted for cell growth analysis or total RNA was collected for qRT-PCR analysis.

### Cell growth and colony formation assay.

Cell proliferation was assessed with a Cell Counting Kit-8 (CCK-8; Dojindo Molecular Technologies, CK04-13) assay, as described by the manufacturer. Briefly, cells were trypsinized, resuspended, and seeded at 1000–1500 cells per well in 96-well plates, where all treatment conditions and time points were in triplicate. The cells were then incubated with 10 μL CCK-8 solution for 2 hours at 37°C and 5% CO_2_. The absorbance was measured at 450 nm using a microplate reader (BioTek Synergy H1). In siRNA-mediated gene silencing experiments, the cells were seeded 48 hours after siRNA transfection. For stable knockdown/knockout experiments based on shRNA/sgRNA, the shRNA/sgRNA-transduced cells were seeded 4 (shRNA) or 10 (sgRNA) days after puromycin selection, respectively. For colony formation assay, shRNA/sgRNA-transduced cells were seeded at 1000 to 1500 cells per well in 6-well plates or 400 to 600 cells per well in 12-well plates, with each treatment condition in triplicate. Medium was changed every day. After 2 weeks, cells were fixed with 100% methanol for 30 minutes and stained with 0.5% crystal violet in PBS for 2 hours. Plates were then washed with distilled water and photographed with a ChemiDoc Touch Imaging System (Bio-Rad). The ColonyArea ImageJ plugin (NIH) was used to calculate colony area percentages.

### Orthotopic xenograft experiments.

Athymic nude mice (7-week-old females, Envigo) were randomly divided into 3 groups (*n* = 6 mice per group). The first group was implanted with ZR75-1 cells stably transduced with EV and shNC; the second group was implanted with ZR75-1 cells stably transduced with EV and shRNA targeting LINC00992; and the third group was implanted with ZR75-1 cells stably transduced with GT3-INCP and LINC00992-targeting shRNA. A total of 4 × 10^6^ ZR75-1 cells were orthotopically injected into the mammary fat pad of mice to study tumor growth. An E2 pellet was implanted under the back of the neck skin to accelerate tumor lesion formation (65). Tumor volume was measured every week for 9 weeks using the formula tumor volume = (*L* × *W*^2^)/2, where *L* represents the largest tumor diameter and *W* represents the perpendicular tumor diameter.

### Data availability.

The sequencing data generated by the current study were deposited in the NCBI Gene Expression Omnibus database (GEO GSE196927). The MS proteomics data were deposited in the ProteomeXchange Consortium via the PRIDE partner repository with the data set identifiers PXD031778 and PXD037137.

### Statistics.

All the experimental data are presented as the mean ± standard deviation (SD). The 2-tailed Student’s *t* test was used for the comparisons between 2 groups, and 1-way ANOVA with Dunnett’s or Tukey’s multiple-comparison test was used for more than 2 groups, using GraphPad Prism 9.0.

### Study approval.

 All mouse experiments were performed according to the NIH *Guide for the Care and Use of Laboratory Animals* (National Academies Press, 2011) and were approved by the Institutional Animal Care and Use Committee (IACUC AN-6813) of the Baylor College of Medicine. The analysis of GT3-INCP protein expression in the fresh-frozen ER^+^ BC tumors and matched normal breast tissues that were collected/banked under protocol PA14-0241 at the University of Texas MDACC was approved by the PA14-0241 Data and Biospecimen Access Committee (DBAC) and MDACC Institutional Review Board (IRB protocol 2022-0245).

## Author contributions

Y Chen conceived the project. CZ, MS, and Y Chen designed the study and analyzed the data. CZ, LX, KL, JH, XL, YD, AJ, and NI conducted experiments. Y Wei, PZ, CZ, and ZZ performed bioinformatics analyses. AM, Y Wu, FD, HX, Y Chiu, and XC contributed to data analysis and interpretation. CZ and Y Chen wrote the manuscript with input from all coauthors. MS, XC, and Y Chen supervised the study.

## Supplementary Material

Supplemental data

Supplemental table 1

Supplemental table 2

Supplemental table 3

Supplemental table 4

Supplemental table 5

Supplemental table 6

Supplemental table 7

Supplemental table 8

Supplemental table 9

Supplemental table 10

## Version 1 03/01/2023

Electronic publication

## Version 2 03/06/2023

Addition of missing graphical abstract
