# Supplementary material for: CRISPR/Cas9 screen uncovers functional translation of cryptic lncRNA-encoded open reading frames in human cancer
Source: J Clin Invest. 2023 Mar 1;133(5):e159940. doi: 10.1172/JCI159940 (PMC9974104; doi:10.1172/JCI159940)
Supplement: Supplemental data [file jci-133-159940-s010.pdf]

Supplementary Methods

Ribosome profiling and library preparation

Sample preparation for ribosome profiling was conducted as described previously[1]. Briefly, MCF7 cells were treated with CHX (Sigma-Aldrich, final concentration 0.1mg/ml) for 1 minute and the cells were lysed by using Mammalian Lysis Buffer (including CHX at a concentration of 0.1mg/ml). Then 600 μL of lysates were taken and 15 μL of RNase I (100 U/ μL, Thermo Fisher Scientific) were added, and the mixtures were incubated for 45min at room temperature followed by adding 15 μL SUPERaseIn RNase inhibitor (Thermo Fisher Scientific) to stop the reaction. Ribosome recovery was done by illustra MicroSpin S-400 HR Columns (GE Healthcare) and the Ribosomal Protected Fragments (PFFs) were purified by RNA Clean & Concentrator (Zymo Research). Ribosomal RNA was subtracted using Ribo-Zero Magnetic Gold Kit (Human/Mouse/Rat, Illumina). RPFs without ribosomal RNA were run on a 15% Urea denaturing-PAGE gel, and the gel slides from 28nt~30nt were excised. The RPF RNAs were eluted and precipitated followed by library construction according to the manufacturer's protocol.

Ribo-seq data analysis and cryptic ORF prediction

The cryptic ORFs were predicted based on Ribo-TISH pipeline[1]. In brief, the ribosome-protected RNA fragments (RPF) reads were trimmed and the low-quality reads were filtered by Sickle (http://github.com/ucdavis-bioinformatics/sickle). The RPF reads after filtering were mapped to human rRNA sequences using bowtie and allowing for two mismatches. The reads that were not mapped to human rRNA sequences were then mapped to the human genome (GRCh38) with transcriptome annotations GENECODE v22, NCBI refseq, MiTranscriptome[2] and lincRNA transcript annotations generated by Dr. John Rinn's group[3], using STAR[4] v.2.6.1b with

parameters “--outFilterMismatchNmax 2 –outFilterIntronMotifs RemoveNoncanonicalUnannotated --alignIntronMax 20000 --outMultimapperOrder Random --outSAMmultNmax 1 --alignEndsType EndToEnd”. Quality control was performed using Ribo-TISH quality module with all the uniquely mapped RPF reads in the annotated ORFs. The RPFs were grouped by their lengths and each aligned RPF read was represented by its 5′ end before estimation of the P-site offset. The metagene RPF count profile near the start/stop codon was constructed by summing the RPF count between −40 and +20 bp of the first base of the start/stop codon across all annotated protein-coding genes. The P-site offset was estimated based on the distribution of the 5′ end of the metagene RPF counts near the annotated start codons. The RPF count between the 15 bp upstream of the first base of the start codon and the 12 bp upstream of the first base of the stop codon were used to calculate the RPF count distributions across three reading frames. The fraction of the RPF counts in the dominant frame (fd) was calculated as the ratio between the maximum RPF count among all three reading frames and the sum of the RPF counts from all reading frames. The cryptic ORFs were predicted with Ribo-TISH predict module with regular riboseq data longest mode. The same ORFs in different ribo-seq libraries and different transcript isoforms were merged, and the predicted cryptic ORFs ($p<0.05$) encoded by lncRNAs with an AUG start codon in either our in-house or publically available ribo-seq data in MCF7 cells (GSE69923)[5] were selected for sgRNA design.

CRISPR-Cas9 sgRNA library design and construction

The sgRNAs targeting the cryptic ORFs that were predicted from ribo-seq data, were designed using the Sequence Scan for CRISPR (SSC) method[6]. The designed sgRNAs that meet one of the following criteria: (1) being mapped to multiple genomic regions; (2)with any Ns or more than

three consecutive T; (3)with high level of GC content (>60%); (4)guide efficiency score< 0.2; (5)being mapped to the annotated CDSs, were filtered out from the library. The 636 sgRNAs targeting 106 core essential genes were included as positive controls, and the 1,064 sgRNAs that target AAVS1 sites in the human genome or do not target the human genome were included as negative controls, respectively. The sgRNAs flanked by linker sequences (Table S10) were synthesized as a pooled library using the CustmoArray 12K chips (CustmoArray, Inc). The array-synthesized sgRNA library was amplified for 8 cycles, using specific primers (Table S10) and Q5 High-Fidelity DNA Polymerase (New England Biolabs #M0491S). The PCR product was purified and assembled into a BsmBI (Thermo Fisher #ER0452)-digested lentiGuide-Puro vector (Addgene #52963) by Gibson assembly (Gibson Assembly® Master Mix, New England Biolabs # E2611L). A total of 2 ul of 10-50 ng/ul ligation products was transfected into 25 ul electrocompetent cells (Lucigen) by using Micropulser Electroporator (Bio-Rad) with one-shot EC1 program (~3-4 reactions for one library). The transformed electrocompetent cells were plated on each of pre-made 24.5 cm2 bioassay plates (ampicillin) using a spreader after recovering in recovery media for 1 hour rotated at 37 °C. All plates were grown inverted for 14 hours at 32 °C. Finally, the colonies were scraped off and the plasmids were extracted with NucleoBond Xtra Midi EF kit (Takara #740422.50) for downstream virus production.

CRISPR-Cas9 screen and data analysis

The MCF7 cells transduced with lentiCas9-EGFP (addgene, #63592) were sorted on a FACSAria cell sorter (BD Biosciences) and the cells with high EGFP expression were collected. These MCF7 cells with high expression of SpCas9 were plated into ten 10-cm dishes and infected with lentiviruses containing the sgRNA library at an MOI of 0.2~0.3. Following

puromycin (2 μg/ml) selection for 4 days, 3.5 $\times 10^7$ cells were split into three replicates. For each replicate, 8× 10^6 were harvested to extract genomic DNAs (Day 0, D0), using QIAamp DNA Mini Kit (QIAGEN), and 3.7×10^6 cells (~500×coverage for each sgRNA per replicate) were passed every 3 days and cultured for 21 days. At day 21 (D21), 8× 10^6 cells were harvested for each replicate to extract genomic DNA. The next-generation-sequencing (NGS)-ready sgRNA libraries were prepared by two rounds of PCR with the KAPA HiFi HotStart ReadyMix (Roche # KK2602). The first-round PCR was conducted for 16 cycles, using 40 ug template of genomic DNA from each replicate at D0 or D21. By using the first-round PCR product as template The second-round PCR was conducted for 12 cycles to incorporate Illumina barcode sequences (Forward: AATGATACGGCGACCACCGAGATCTACAC<Illumina index 8-nt barcode > ACACTCTTTCCCTACACGACGCTCTTCCGATCTTCTTGTGGAAAGGACGAAACACCG; Reverse: CAAGCAGAAGACGGCATACGAGAT<Illumina index 8-nt barcode > GTGACTGGAGTTCAGACGTGTGCTCTTCCGATCTCTACTATTCTTTCCCCTGCACTGT ACC). The final PCR product was purified from 2% agarose gel with QIAquick Gel Extraction Kit. Concentration of different libraries was measured using the Qubit dsDNA HS (High Sensitivity) Assay Kit (Thermo # on a Qubit Fluorometer (Thermo fisher). The libraries were pooled with equal proportion were sequenced on an Illumina NextSeq 500 for single read 76 cycles, at the Advanced Technology Genomics Core of MDACC. MAGeCK[7] (v0.5.9.4) was used to calculate the read count of individual sgRNAs in different samples with the following parameters: "mageck count -l sgrna.library --control-sgrna sgrna.library.negctrl --norm-method control -n sgrna.count --sample-label D0,D21 --fastq files.fq". DESeq2[8] was used to identify the differentially expressed sgRNAs between D0 and D21. The read counts were normalized by the mapped negative control sgRNAs using ratio median normalization, and the normalization

factors were applied to all sgRNAs. The cryptic ORFs with at least 2 significantly depleted sgRNAs ($\log_2$(Fold-Change)<−$\log_2$(1.5) and p<0.05), whose expression was up-regulated in Luminal A BRCA than normal breast tissues. ($\log_2$(Fold-Change)≥$\log_2$(1.2), *FDR*<0.01) were selected as the final candidates of Luminal A BRCA dependency.

5' and 3' RACE

The 5' and 3' RACE experiments were conducted using the SMARTer® RACE 5'/3' Kit (Clontech #634859) as described previously[9]. Total RNA from MCF7 cells was extracted using the RNeasy Mini kit (QIAGEN #74104) according to the manufacturer's instruction. First-strand cDNA was synthesized using 5′-CDS and 3′-CDS primer A and SMARTer II A oligonucleotide as described in the user's manual. The touchdown nested PCR was used to amplify cDNA ends. All the primers are listed in Supplementary Table 10. The PCR product was purified from 2% agarose gel with NuceloSpin Gel and PCR Clean-Up Kit (supplied with the SMARTer® RACE 5'/3' Kit) and was then cloned into pRACE vector using In-Fusion HD Master Mix (both vector and mix were provided as SMARTer RACE 5'/3' Kit Components) for Sanger sequencing.

RNA-seq

Total RNA was isolated from cells using RNeasy Mini kit (QIAGEN, #74104) and was treated with DNase I(QIAGEN #79254). RNA-seq libraries were prepared from 3 µg of total RNA, using TruSeq Stranded mRNA Library Prep kit (Illumina # 20020594), according to the manufacturer's instructions. The libraries were sequenced on an Illumina NextSeq 500 (single-end 76-bp), at the Advanced Technology Genomics Core of MDACC.

RNA-seq/ChIP-seq data analysis, integrative analyses of TCGA data and breast cancer susceptibility/risk gene curation

The RNA-seq and ChIP-seq reads were first trimmed by Trim Galore (v0.6.5) (https://www.bioinformatics.babraham.ac.uk/projects/trim_galore/), a wrapper around two tools: cutadapt v2.8 (https://github.com/marcelm/cutadapt/) and FastQC v0.11.5 (https://github.com/chgibb/FastQC0.11.5/; https://www.bioinformatics.babraham.ac.uk/projects/fastqc/), and were then mapped to the human genome (GRCh38), using HISAT2[10](v2.1.0) and Bowtie 2[11] (v2.4.1), respectively. For RNA-seq, The gene-level raw read-counts were calculated using htseq-count function of HTSeq[12] (0.11.0), based on the aligned and sorted bam files. The normalization of read counts and differential gene expression analysis were performed, using DESeq2[8](1.22.2). The filters of basemean>1, $|\log_2(\text{Fold-Change})| \geq \log_2(1.5)$ and $FDR<0.05$ were used to define differentially expressed genes for most downstream analysis. The GATA3 ChIP-seq data generated from MC7 and T47D cells were obtained from ENCODE (GSE32465) and GSE128460. The sorted BAM files were converted into bedGraph and bigWig formats using BEDTools[13] (v2.24.0) and UCSC bedGraphToBigWig[14] (v4). The ChIP-seq peaks were identified by MACS2[15] (v2.1.2) with the parameters "macs2 callpeak -t ChIP.bam -c INPUT.bam -g hs --outdir output -n NAME 2> NAME.callpeak.log" for GATA3 and GT3-INCP ChIP-seq data. The genome-wide distribution of GT3-INCP binding sites was calculated using the CEAS tool in Cistrome[16], a web-based analysis platform for transcriptional regulation studies. The conservation plot of the GT3-INCP binding sites and their flanking sequences was made with a window size of +/- 500 bps in Cistrome. The motif enrichment analysis was performed using the Cistrome SeqPos tool. The BETA (1.0.7)[17] was used to find the target genes of the ChIP-seq peaks. The gene ontology

enrichment analysis and KEGG pathway enrichment analysis was performed by DAVID[18], with the genes co-regulated by LINC00992/GT3-INCP and GATA3 (basemean>1, $|\log_2$(Fold-Change)$| \geq \log_2(1.2)$ and $FDR<0.05$) being the input gene list. Gene Set Enrichment Analysis[19] (GSEA) was performed using the hallmark gene sets from the Molecular Signatures Database[20], with GSEA (v4.2.2) Java desktop program. TCGA breast cancer (BRCA) RNA-seq read count data were downloaded from TCGA. Raw count normalization, and differential gene expression analysis between tumors and the corresponding normal breast tissues were performed using DESeq2. The genes with "basemean>1, $\log_2$(Fold-Change)$\geq \log_2(1.2)$ and $FDR<0.01$" were considered as the significantly up-regulated genes in tumor vs. normal. The Kaplan-Meier survival curves were used to show the survival distributions and the log-rank test was used to assess the corresponding statistical significance. The survival analysis was performed, using the "survival" and "survminer" package in R. Breast cancer susceptibility/risk genes were compiled from a total of 32 published studies based on GWAS, cis-QTL and/or integrative analysis of functional genomic data (Table S7).

Generation of custom anti-GT3-INCP rabbit polyclonal antibody

The custom anti-GT3-INCP rabbit polyclonal antibody was generated by ABclonal Technology. Briefly, 1.5 year old New Zealand rabbits with weight of 2.5 kg under specific-pathogen-free conditions were injected subcutaneously with 300 μg purified antigen protein (full-length 120 aa GT3-INCP) supplemented with Complete Freund's Adjuvant (CFA) for the primary injection and 150 μg antigen protein supplemented with Incomplete Freund's Adjuvant (IFA) for three subsequent boosting injections at 2 weeks intervals. Terminal bleeds were collected after immunization and rabbit polyclonal antibodies were purified from terminal bleeds by antigen

affinity chromatography.

Three-dimensional structure prediction

The three-dimensional protein structure of the full-length GATA3 was obtained from Alphafold Database[21,22] or predicted by I-TASSER-MTD webserver (https://zhanggroup.org/I-TASSER-MTD)[23,24] with default parameters. Five structural models were predicted by I-TASSER-MTD webserver, among which the model that showed the best alignment with the experimentally determined structures of ZF1 and ZF2 domain (PDB ID: 4hc7) was presented. The structures of the GT3-INCP protein were predicted by I-TASSER webserver (https://zhanggroup.org/I-TASSER/)[25,26] with default parameters and Alphafold2[21,27], respectively.

ChIP-qPCR and ChIP-seq

ChIP was performed as described in Duncan Odom's group's protocol with some adaptations[28]. In brief, at about 80-90% confluence, approximately 2×10^7 MCF7 or T47D cells were first crosslinked with 1% formaldehyde (methanol-free, 16% Thermo Scientific, #28908) at room temperature for 10 minutes and then quenched with 0.125M Glycine (final concentration) for 5 minutes. For GT3-INCP ChIP experiments, the MCF7 cells stably expressing FLAG-tagged GT3-INCP were used. After washing with cold PBS for three times, the cells were harvested using a silicon scraper. Cell pellets were resuspended in 5mL lysis buffer 1(50 mM Hepes-KOH, pH 7.5, 140 mM NaCl, 1 mM EDTA, 10% glycerol, 0.5% NP-40, 0.25% Triton X-100) and rocked at 4 degree for 5 minutes, followed by centrifugation at 2000 g for 4 minutes at 4°C. The cell pellets were then incubated with 5 mL LB2 buffer (10 mM Tris-HCl, pH=8.0, 200 mM NaCl, 1 mM EDTA, 0.5 mM EGTA) at 4°C for 5 minutes with gently rocking. Nuclei were pelleted

down by centrifugation of the cells at 2000 g for 5 min and were resuspended in 1 mL LB3 buffer (10 mM Tris-HCl, pH=8.0, 100 mM NaCl, 1 mM EDTA, 0.5 mM EGTA, 0.1% Na-Deoxycholate, 0.5% N-Lauroylsarcosine). All lysis buffer contained protease inhibitors (Roche #04693112001). Chromatin was sonicated to around 200 bp DNA fragments, using a Diagenode Bioruptor (three rounds of 5 cycles, 30" on 30" off). Lysates were cleared by addition of Triton X-100 to a final concentration of 1% and centrifugation at 2000 g for 10 minutes at 4°C. A total of 50 µl lysates were saved from each sample for input and stored at – 80°C until use. To prepare antibody-bound beads, 30 µl of magnetic beads (Invitrogen, Dynabeads) were washed three times with blocking buffer (1× PBS, 0.5% BSA) and incubated overnight with 5 µg of anti-GATA3 or anti-FLAG antibody at 4°C. For each ChIP, 900 uL sonicated lysate from 2×10^7 cells was incubated with the antibody-bound beads overnight at 4°C. Beads were washed 6 times with RIPA wash buffer (50 mM Hepes-KOH, pH= 7.6, 500 mM LiCl, 1 mM EDTA, 1% NP-40, 0.7% Na-Deoxycholate) and 1 time with TBS (20 Mm Tris-HCL, PH 7.6, 150Mm NaCl) for 5 minutes each time at room temperature with gently rocking. All washing buffers contain the protease inhibitors. The beads were eluted twice with 50 ul elution buffer (50 mM Tris-HCl pH 8, 10 mM EDTA, 1% SDS) for 10 minutes at 65°C with rocking. Crosslinking was reversed by adding 6 uL 5M NaCl to the eluates and incubated at 65°C overnight. RNAs were degraded by incubation with 1µl of 10 mg/ml RNase at 37°C for 30 minutes and proteins were digested by incubation with 2 µl of 20 mg/ml of Proteinase K(Thermo Fisher) at 56°C for 2 hours. DNAs were then purified using QIAquik PCR purification kit (QIAGEN, #28106). The samples were analyzed by qPCR or further processed for sequencing. ChIP-seq libraries were prepared from 10 ng of ChIP DNA using the TruSeq ChIP Library Preparation Kit (Illumina, # IP-202-1012), according to the

manufacturer's instructions. The libraries were sequenced on an Illumina NextSeq 500 (single-end 76-bp), at the Advanced Technology Genomics Core of MDACC.

Immunoprecipitation, subcellular fractionation and western blotting

For immunoprecipitation assays, the cells were lysed in Pierce IP lysis buffer (Thermo Fisher, #87787) with protease inhibitor and 10mM PMSF (Thermo Fisher, #36978). For immunoprecipitation of exogenous FLAG-tagged proteins, anti-FLAG M2 agarose Beads (Sigma-Aldrich, #A2220) were incubated with the whole cell lysates overnight with gently rotating at 4 °C. For immunoprecipitation of endogenous proteins, the specific antibodies were first coupled to protein G magnetic beads (Invitrogen, #10004D) and then incubated with the cell lysates. After incubation, the beads were washed 5 times with washing buffer (10 mM Tris, PH 7.4, 1 mM EDTA, 1 mM EGTA, pH 8.0, 150 mM NaCl, 1% Triton X-100) and resuspended in SDS-PAGE sample buffer(Bio-Rad #1610747). For mass spectrometry, the precipitated proteins on the beads were eluted by a competition with 3×FLAG peptides (Sigma-Aldrich, #F4799). Eluted proteins and 5% of the whole-cell extracts were analyzed by immunoblot. To confirm the interaction between GT3-INCP and GATA3 on chromatin, the immunoprecipitation from chromatin extracts was performed as described previously[29,30], which is similar to the ChIP experiment. After 6 times of RIPA washing, the beads were re-suspended in SDS-PAGE sample buffer (Bio-Rad #1610747) for western blot analysis. To segregate and enrich nuclear and cytoplasmic proteins, the subcellular protein fractionation kit for cultured cells (Thermo Scientific™, 78840) was used for ER+ cell lines, according to the manufacturer's instructions Whole-cell lysates were generated using RIPA lysis and extraction buffer (Thermo Fisher #89900) supplemented with protease Inhibitor Cocktail (Sigma #11697498001) according to the

manufacturer's instructions. Protein concentration was measured by using the Bradford assay (Bio-Rad # 5000006). Proteins were separated by 4-15% or 4-20% Mini-PROTEAN TGX precast polyacrylamide gel (Bio-Rad), and then transferred to PVDF membranes (Millipore, #GVWP04700) in a transfer buffer (Invitrogen, #LC3675) at 4 °C. Membranes were first blocked and incubated with specific antibodies overnight at 4 °C, and then incubated with immobilon western chemiluminescent HRP substrate (Millipore, #WBKLS0500) followed by analysis using ChemiDoc Touch Imaging Systems (Bio-Rad).

Immunofluorescence staining

The MCF7 cells stably expressing FLAG-tagged GT3-INCP were seeded into 4-well culture/chamber slides (Lab-Tek, 154917) with 30-50% confluency. Cells were washed with cold PBS and fixed using 4% paraformaldehyde for 15 min followed by permeabilization in 0.25% Triton X-100 solution for 10 min at room temperature. The fixed cells were blocked with 10% normal goat serum (Life Technologies, PCN5000) in PBS for 30 min at room temperature, and then incubated with anti-FLAG antibody (Sigma, F1804) at 1:500 in PBS overnight at 4°C. After washing, the cells were incubated with fluorochrome-conjugated secondary antibody (Invitrogen, A32723) at 1:1000 in PBS for 1 hour at room temperature in dark. The slips were mounted onto the microscope slide with Vectashield Mounting Medium containing DAPI (Vector Laboratories, H-1500). The images were captured by ZEISS LISM880 confocal microscopy.

References

1. Zhang, P. *et al.* Genome-wide identification and differential analysis of translational initiation. *Nat Commun* **8**, 1749 (2017).
2. Iyer, M.K. *et al.* The landscape of long noncoding RNAs in the human transcriptome. *Nat Genet* **47**, 199-208 (2015).
3. Cabili, M.N. *et al.* Integrative annotation of human large intergenic noncoding RNAs reveals global properties and specific subclasses. *Genes Dev* **25**, 1915-27 (2011).
4. Dobin, A. *et al.* STAR: ultrafast universal RNA-seq aligner. *Bioinformatics* **29**, 15-21 (2013).
5. Loayza-Puch, F. *et al.* Tumour-specific proline vulnerability uncovered by differential ribosome codon reading. *Nature* **530**, 490-4 (2016).
6. Xu, H. *et al.* Sequence determinants of improved CRISPR sgRNA design. *Genome Res* **25**, 1147-57 (2015).
7. Li, W. *et al.* MAGeCK enables robust identification of essential genes from genome-scale CRISPR/Cas9 knockout screens. *Genome Biol* **15**, 554 (2014).
8. Love, M.I., Huber, W. & Anders, S. Moderated estimation of fold change and dispersion for RNA-seq data with DESeq2. *Genome Biol* **15**, 550 (2014).
9. Sun, M. *et al.* Systematic functional interrogation of human pseudogenes using CRISPRi. *Genome Biology* **22**, 240 (2021).
10. Kim, D., Paggi, J.M., Park, C., Bennett, C. & Salzberg, S.L. Graph-based genome alignment and genotyping with HISAT2 and HISAT-genotype. **37**, 907-915 (2019).
11. Langmead, B. & Salzberg, S.L. Fast gapped-read alignment with Bowtie 2. *Nat Methods* **9**, 357-9 (2012).
12. Anders, S., Pyl, P.T. & Huber, W. HTSeq--a Python framework to work with high-throughput sequencing data. *Bioinformatics* **31**, 166-9 (2015).
13. Quinlan, A.R. & Hall, I.M. BEDTools: a flexible suite of utilities for comparing genomic features. *Bioinformatics* **26**, 841-2 (2010).
14. Kent, W.J., Zweig, A.S., Barber, G., Hinrichs, A.S. & Karolchik, D. BigWig and BigBed: enabling browsing of large distributed datasets. *Bioinformatics* **26**, 2204-7 (2010).
15. Zhang, Y. *et al.* Model-based analysis of ChIP-Seq (MACS). *Genome Biol* **9**, R137 (2008).
16. Liu, T. *et al.* Cistrome: an integrative platform for transcriptional regulation studies. *Genome Biol* **12**, R83 (2011).
17. Wang, S. *et al.* Target analysis by integration of transcriptome and ChIP-seq data with BETA. *Nat Protoc* **8**, 2502-15 (2013).
18. Dennis, G., Jr. *et al.* DAVID: Database for Annotation, Visualization, and Integrated Discovery. *Genome Biol* **4**, P3 (2003).
19. Subramanian, A. *et al.* Gene set enrichment analysis: a knowledge-based approach for interpreting genome-wide expression profiles. *Proc Natl Acad Sci U S A* **102**, 15545-50 (2005).
20. Liberzon, A. *et al.* The Molecular Signatures Database (MSigDB) hallmark gene set collection. *Cell Syst* **1**, 417-425 (2015).
21. Jumper, J. *et al.* Highly accurate protein structure prediction with AlphaFold. *Nature* **596**, 583-589 (2021).
22. Varadi, M. *et al.* AlphaFold Protein Structure Database: massively expanding the structural coverage of protein-sequence space with high-accuracy models. *Nucleic Acids Res* **50**, D439-d444 (2022).
23. Zhou, X., Hu, J., Zhang, C., Zhang, G. & Zhang, Y. Assembling multidomain protein structures through analogous global structural alignments. *Proc Natl Acad Sci U S A* **116**, 15930-15938 (2019).

24. Zhou, X. *et al.* I-TASSER-MTD: a deep-learning-based platform for multi-domain protein structure and function prediction. *Nat Protoc* **17**, 2326-2353 (2022).
25. Zheng, W. *et al.* Folding non-homologous proteins by coupling deep-learning contact maps with I-TASSER assembly simulations. *Cell Rep Methods* **1**(2021).
26. Yang, J. & Zhang, Y. I-TASSER server: new development for protein structure and function predictions. *Nucleic Acids Res* **43**, W174-81 (2015).
27. Mirdita, M. *et al.* ColabFold: making protein folding accessible to all. *Nat Methods* **19**, 679-682 (2022).
28. Schmidt, D. *et al.* ChIP-seq: using high-throughput sequencing to discover protein-DNA interactions. *Methods* **48**, 240-8 (2009).
29. Mohammed, H. *et al.* Rapid immunoprecipitation mass spectrometry of endogenous proteins (RIME) for analysis of chromatin complexes. *Nat Protoc* **11**, 316-26 (2016).
30. Mohammed, H. *et al.* Endogenous purification reveals GREB1 as a key estrogen receptor regulatory factor. *Cell Rep* **3**, 342-9 (2013).

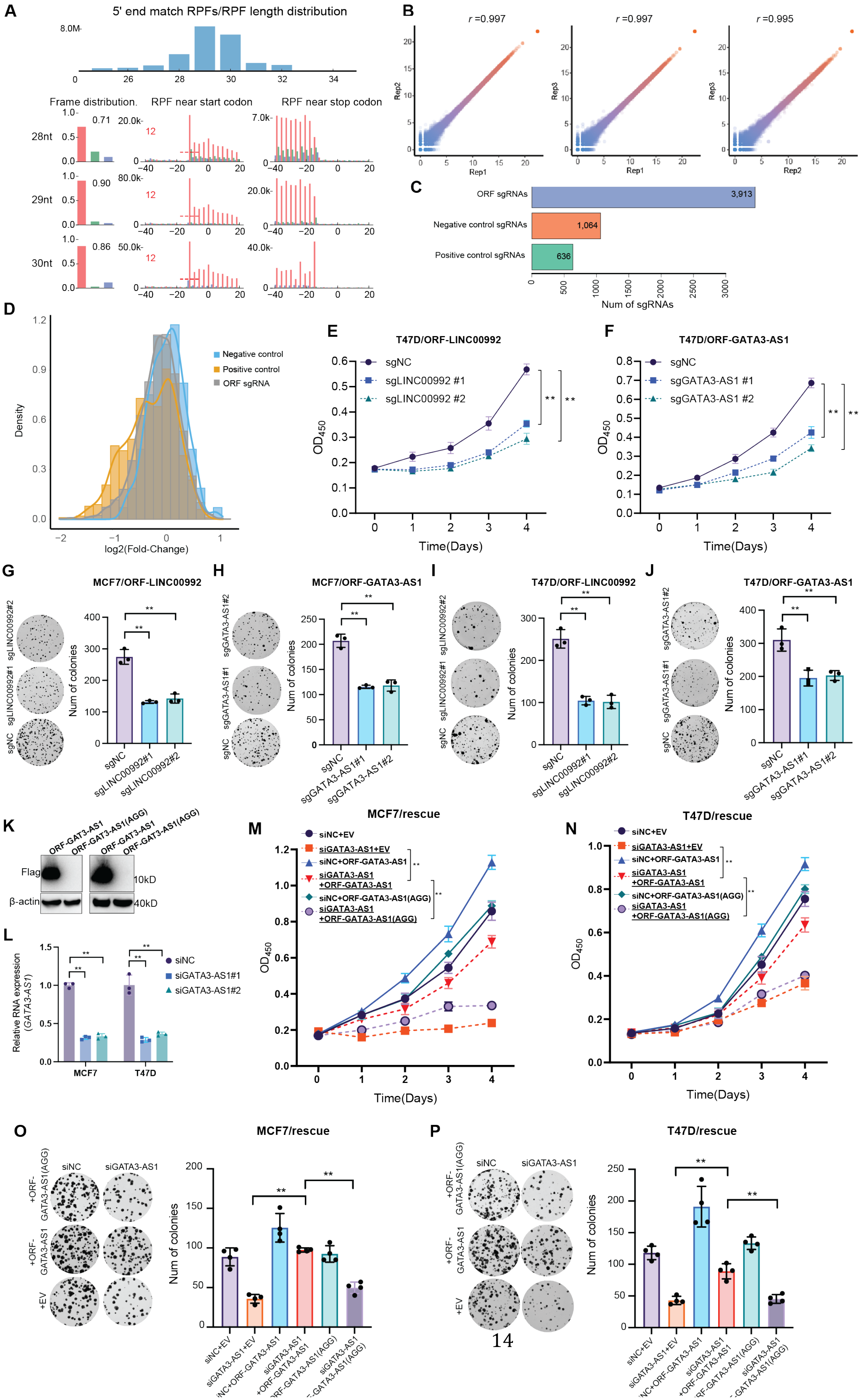
A
5' end match RPFs/RPF length distribution
8.0M
0 26 28 30 32 34
Frame distribution.
RPF near start codon
RPF near stop codon
28nt
29nt
30nt
0.71
0.90
0.86
20.0k
80.0k
50.0k
7.0k
20.0k
40.0k
12
B
r =0.997
r =0.997
r =0.995
Rep2
Rep1
Rep3
Rep1
Rep3
Rep2
C
ORF sgRNAs
3,913
Negative control sgRNAs
1,064
Positive control sgRNAs
636
Num of sgRNAs
D
Density
log2(Fold-Change)
Negative control
Positive control
ORF sgRNA
E
T47D/ORF-LINC00992
sgNC
sgLINC00992 #1
sgLINC00992 #2
OD450
Time(Days)
F
T47D/ORF-GATA3-AS1
sgNC
sgGATA3-AS1 #1
sgGATA3-AS1 #2
OD450
Time(Days)
G
MCF7/ORF-LINC00992
sgNC
sgLINC00992#1
sgLINC00992#2
Num of colonies
H
MCF7/ORF-GATA3-AS1
sgNC
sgGATA3-AS1#1
sgGATA3-AS1#2
Num of colonies
I
T47D/ORF-LINC00992
sgNC
sgLINC00992#1
sgLINC00992#2
Num of colonies
J
T47D/ORF-GATA3-AS1
sgNC
sgGATA3-AS1#1
sgGATA3-AS1#2
Num of colonies
K
ORF-GAT3-AS1
ORF-GAT3-AS1(AGG)
ORF-GAT3-AS1
ORF-GAT3-AS1(AGG)
Flag
β-actin
10kD
40kD
L
Relative RNA expression (GATA3-AS1)
siNC
siGATA3-AS1#1
siGATA3-AS1#2
MCF7
T47D
M
MCF7/rescue
siNC+EV
siGATA3-AS1+EV
siNC+ORF-GATA3-AS1
siGATA3-AS1 +ORF-GATA3-AS1
siNC+ORF-GATA3-AS1(AGG)
siGATA3-AS1 +ORF-GATA3-AS1(AGG)
OD450
Time(Days)
N
T47D/rescue
siNC+EV
siGATA3-AS1+EV
siNC+ORF-GATA3-AS1
siGATA3-AS1 +ORF-GATA3-AS1
siNC+ORF-GATA3-AS1(AGG)
siGATA3-AS1 +ORF-GATA3-AS1(AGG)
OD450
Time(Days)
O
MCF7/rescue
siNC
siGATA3-AS1
+ORF-GATA3-AS1(AGG)
+ORF-GATA3-AS1
+EV
Num of colonies
siNC+EV
siGATA3-AS1+EV
siNC+ORF-GATA3-AS1
siGATA3-AS1 +ORF-GATA3-AS1
siNC+ORF-GATA3-AS1(AGG)
siGATA3-AS1 +ORF-GATA3-AS1(AGG)
P
T47D/rescue
siNC
siGATA3-AS1
+ORF-GATA3-AS1(AGG)
+ORF-GATA3-AS1
+EV
Num of colonies
siNC+EV
siGATA3-AS1+EV
siNC+ORF-GATA3-AS1
siGATA3-AS1 +ORF-GATA3-AS1
siNC+ORF-GATA3-AS1(AGG)
siGATA3-AS1 +ORF-GATA3-AS1(AGG)

Figure S1. (A) Representative quality control of ribo-seq data. Upper panel: length distribution of the ribosome-protected fragments or footprints (RPFs) uniquely mapped to the annotated protein-coding regions. Lower panel: different quality profiles/metrics for RPFs uniquely mapped to the annotated protein-coding regions. Each row shows the RPFs with indicated length. Column 1: the distribution of RPF count across 3 reading frames across the annotated codons; showing the percentage of reads from the dominant reading frame. Column 2: distribution of RPF count near the annotated TISs; showing estimated P-site offset and TIS accuracy. Column 3: distribution of RPF count near the annotated stop codons. **(B)** The scatter plot showing the correlation between three replicates of ribo-seq data. **(C)** Bar graph showing the number of sgRNAs targeting the cryptic lncRNA-encoded ORFs identified from ribo-seq data in MCF7, and the number of positive and negative control sgRNAs. **(D)** The histograms showing the distribution of log_2(Fold-Change) between day 21 and day 0 for sgRNAs targeting the cryptic ORFs (grey), the positive (orange) and negative control sgRNAs (blue) in the CRISPR/Cas9 screen. The growth of the T47D cells transduced with the negative control sgRNA (sgNC)/gene-specific sgRNAs targeting the **(E)** ORF-LINC00992 or **(F)** ORF-GATA3-AS1, was monitored with CCK-8 assay. The OD450 absorbance for WST-8 formazan was measured each day for 4 days. The representative pictures of clonogenic growth and the bar graph quantifying the colonies formed by the MCF7 cells that were transduced with the sgNC/sgRNAs targeting the **(G)** ORF-LINC00992 or **(H)** ORF-GATA3-AS1, after the cells were cultured for two weeks. The representative pictures of clonogenic growth and the bar graph quantifying the colonies formed by the T47D cells that were transduced with the sgNC/sgRNAs targeting the **(I)** ORF-LINC00992 or **(J)** ORF-GATA3-AS1. **(K)** The wild-type FLAG-tagged ORF-GATA3-AS1 or the mutant one (AGG mutation in start codon) was stably expressed in MCF7 and T47D cells and the protein expression was determined by western blot with an anti-FLAG antibody, where β-actin was used as a loading control. **(L)** QRT-PCR was performed to determine the siRNA-mediated GATA3-AS1 knockdown efficiency in MCF7 and T47D cells, where GAPDH served as an internal control. The rescue experiment results for the cell growth defect caused by GATA3-AS1 knockdown are shown. The **(M)** MCF7 and **(N)** T47D cells stably transduced with the ORF-GATA3-AS1 that has a wild-type (ATG)/mutant (AGG) start codon or the empty vector control (EV), were transfected with the negative control siRNA (siNC) or the siRNAs targeting GATA3-AS1 outside the CDS region (siGATA3-AS1) and were cultured for 4 days. The cell growth was monitored each day with CCK-8 assay. The rescue experiment results for the clonogenic growth defect caused by GATA3-AS1 knockdown are shown. The representative pictures of clonogenic growth and the bar graph quantifying the colonies formed by the **(O)** MCF7 or **(P)** T47D cells that were transduced with the wild-type/mutant (AGG mutation in start codon) ORF-GATA3-AS1 or the EV control, and were transfected with the siNC and the siRNAs targeting GATA3-AS1. Data (**E-J** and **L-P**) are shown as mean+/-standard deviation (SD), n=3. One-way ANOVA with Dunnett's multiple comparison test (*$P<0.05$; **$P<0.01$; ns: not significant, $P>0.05$). Data (**K**) are representative of 3 independent experiments.

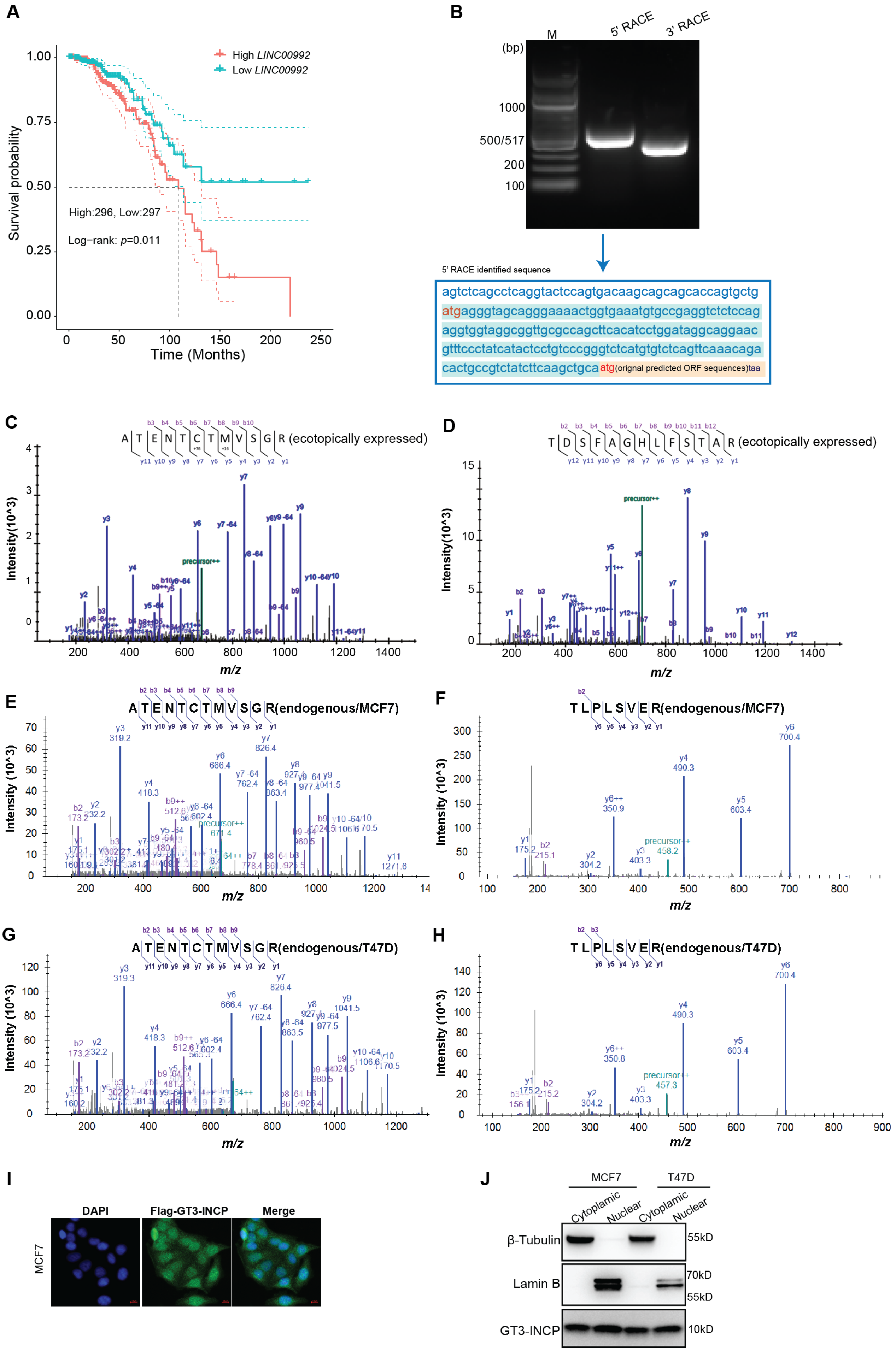
A
High LINC00992
Low LINC00992
Survival probability
High:296, Low:297
Log−rank: p=0.011
Time (Months)
B
M
5' RACE
3' RACE
(bp)
1000
500/517
200
100
5' RACE identified sequence
agtctcagcctcaggtactccagtgacaagcagcagcaccagtgctg
atgagggtagcagggaaaactggtgaaatgtgccgaggtctctccag
aggtggtaggcggttgcgccagcttcacatcctggataggcaggaac
gtttccctatcatactcctgtcccgggtctcatgtgtctcagttcaaacaga
cactgccgtctatcttcaagctgcaatg(orignal predicted ORF sequences)taa
C
A T E N T C T M V S G R (ecotopically expressed)
Intensity(10^3)
m/z
D
T D S F A G H L F S T A R (ecotopically expressed)
Intensity(10^3)
m/z
E
A T E N T C T M V S G R(endogenous/MCF7)
Intensity (10^3)
m/z
F
T L P L S V E R(endogenous/MCF7)
Intensity (10^3)
m/z
G
A T E N T C T M V S G R(endogenous/T47D)
Intensity (10^3)
m/z
H
T L P L S V E R(endogenous/T47D)
Intensity (10^3)
m/z
I
DAPI
Flag-GT3-INCP
Merge
MCF7
J
MCF7
T47D
Cytoplamic
Nuclear
Cytoplamic
Nuclear
β-Tubulin
55kD
Lamin B
70kD
55kD
GT3-INCP
10kD

Figure S2. (A) Higher LINC00992 RNA expression was associated with worse overall survival of the patients with luminal tumors, based on TCGA data. The Kaplan-Meier survival curves are plotted for patient groups with high (top 50%) and low (bottom 50%) LINC00992 RNA expression in luminal tumors. The *p*-value was calculated using log-rank test. **(B)** The 5' and 3'end of the LINC00992 transcript (ENST00000504107.1) were identified by 5' and 3' RACE. An extension of the 5' end was identified compared with the transcript annotation from GENECODE v22, whereas the 3'end was the same as the annotated one. **(C, D)** The MS2 spectra of the two GT3-INCP-derived tryptic peptides that were detected by MS in the proteins co-IPed with an anti-FLAG antibody from the lysates of the MCF7 cells ectopically expressing FLAG-tagged GT3-INCP. The MS2 spectra of the two GT3-INCP-derived tryptic peptides detected by PRM-MS in the proteins co-IPed with an anti-GT3-INCP antibody from the lysates of the (**E**,**F**) MCF7 and (**G**,**H**) T47D cells. (**I**) The sub-cellular localization of the FLAG-tagged GT3-INCP was determined by the immunofluorescence staining with an anti-FLAG antibody in the MCF7 cells stably expressing FLAG-tagged GT3-INCP, where cell nuclei were stained with DAPI. **(J)** The endogenous expression of GT3-INCP in the cytoplasmic and nuclear fraction of MCF7/T47D cells was detected by western blot, where β-tubulin and lamin B served as cytoplasmic and nuclear marker, respectively. (**B** and **I-J**) Data are representative of 3 independent experiments.

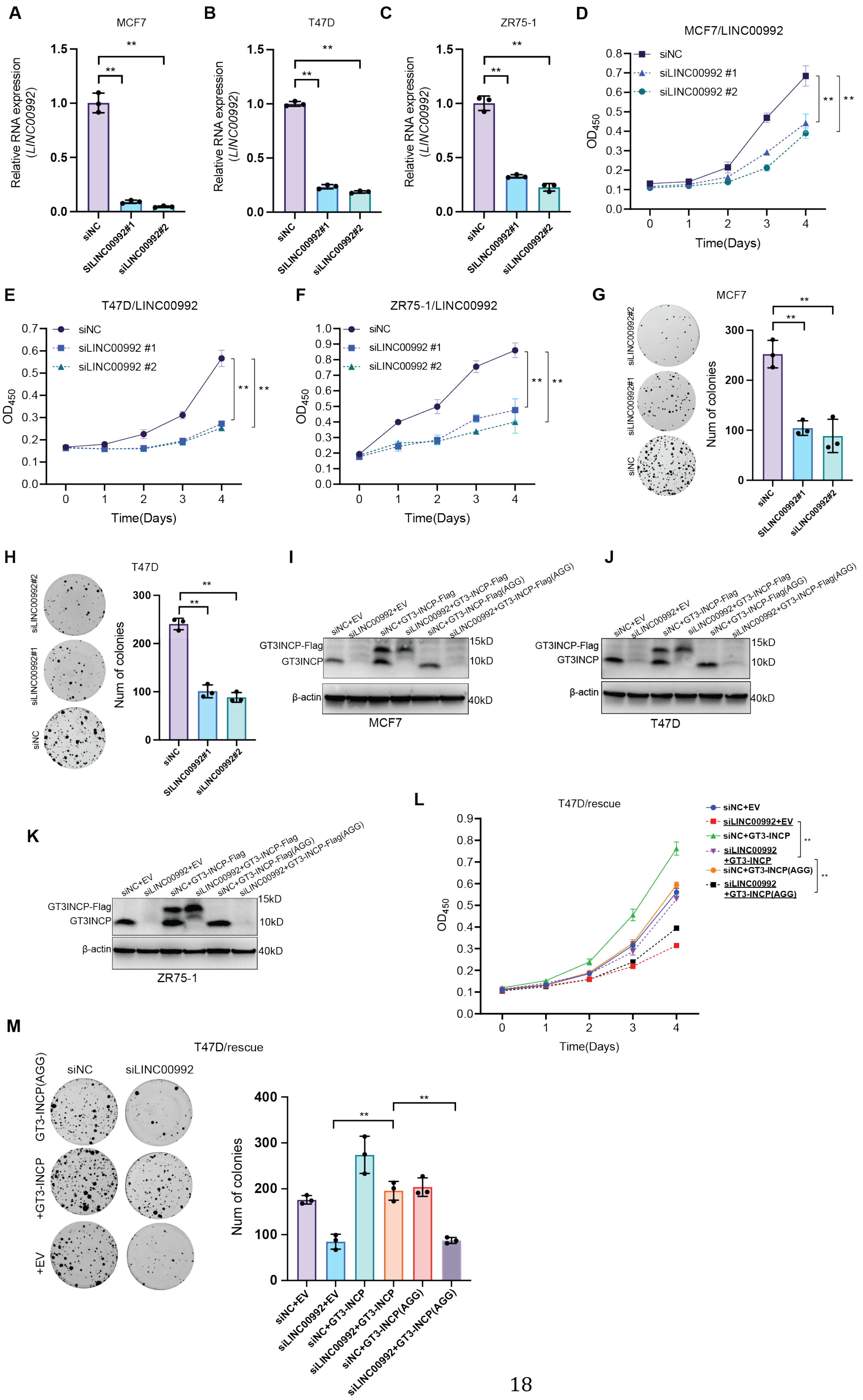
A
MCF7
Relative RNA expression (LINC00992)
siNC
siLINC00992#1
siLINC00992#2
B
T47D
C
ZR75-1
D
MCF7/LINC00992
siNC
siLINC00992 #1
siLINC00992 #2
OD450
Time(Days)
E
T47D/LINC00992
F
ZR75-1/LINC00992
G
MCF7
Num of colonies
H
T47D
I
siNC+EV
siLINC00992+EV
siNC+GT3-INCP-Flag
siLINC00992+GT3-INCP-Flag
siNC+GT3-INCP-Flag(AGG)
siLINC00992+GT3-INCP-Flag(AGG)
GT3INCP-Flag
GT3INCP
β-actin
15kD
10kD
40kD
MCF7
J
T47D
K
ZR75-1
L
T47D/rescue
siNC+EV
siLINC00992+EV
siNC+GT3-INCP
siLINC00992 +GT3-INCP
siNC+GT3-INCP(AGG)
siLINC00992 +GT3-INCP(AGG)
M
T47D/rescue
siNC
siLINC00992
GT3-INCP(AGG)
+GT3-INCP
+EV
siNC+EV
siLINC00992+EV
siNC+GT3-INCP
siLINC00992+GT3-INCP
siNC+GT3-INCP(AGG)
siLINC00992+GT3-INCP(AGG)

Figure S3. QRT-PCR was performed to determine the siRNA-mediated LINC00992 knockdown efficiency in **(A)** MCF7, **(B)** T47D, and **(C)** ZR75-1 cells. The growth of the **(D)** MCF7, **(E)**T47D and **(F)** ZR75-1 cells transfected with the negative control siRNA (siNC) or individual siRNAs targeting LINC00992 was monitored with CCK-8 assay each day for 4 days. The representative pictures of clonogenic growth and the bar graph quantifying the colonies formed by the **(G)** MCF7 and **(H)** T47D cells that were transfected with the siNC or the LINC00992-targeting siRNAs. The **(I)** MCF7, **(J)** T47D and **(K)** ZR75-1 cells stably transduced with the GT3-INCP that has a wild-type (ATG)/mutant (AGG) start codon or the empty vector control (EV), were transfected with the siNC or siRNAs targeting LINC00992. The expression of endogenous GT3-INCP and the ectopically expressed FLAG-tagged GT3-INCP was determined by western blot with an anti-GT3-INCP antibody. **(L)** The rescue experiment results for the cell growth defect caused by siRNA-mediated LINC00992 depletion in T47D cells are shown. The T47D cells stably transduced with the wild-type/mutant (AGG mutation in start codon) GT3-INCP or the EV, were transfected with the siNC or siRNAs targeting LINC00992 and were cultured for 4 days. The cell growth was monitored each day with CCK-8 assay. **(M)** The rescue experiment results for the clonogenic growth defect caused by LINC00992 knockdown in T47D cells are shown. The representative pictures of clonogenic growth and the bar graph quantifying the colonies formed by the T47D cells that were transduced with the wild-type/mutant (AGG mutation in start codon) GT3-INCP or the EV control, and were transfected with the siNC and the siRNAs targeting LINC00992. Data (**A**-**H** and **L**-**M**) are shown as mean+/-standard deviation (SD), n=3. One-way ANOVA with Dunnett's multiple comparison test (*$P<0.05$; **$P<0.01$; ns: not significant, $P>0.05$). Data (**I**-**K**) are representative of 3 independent experiments.

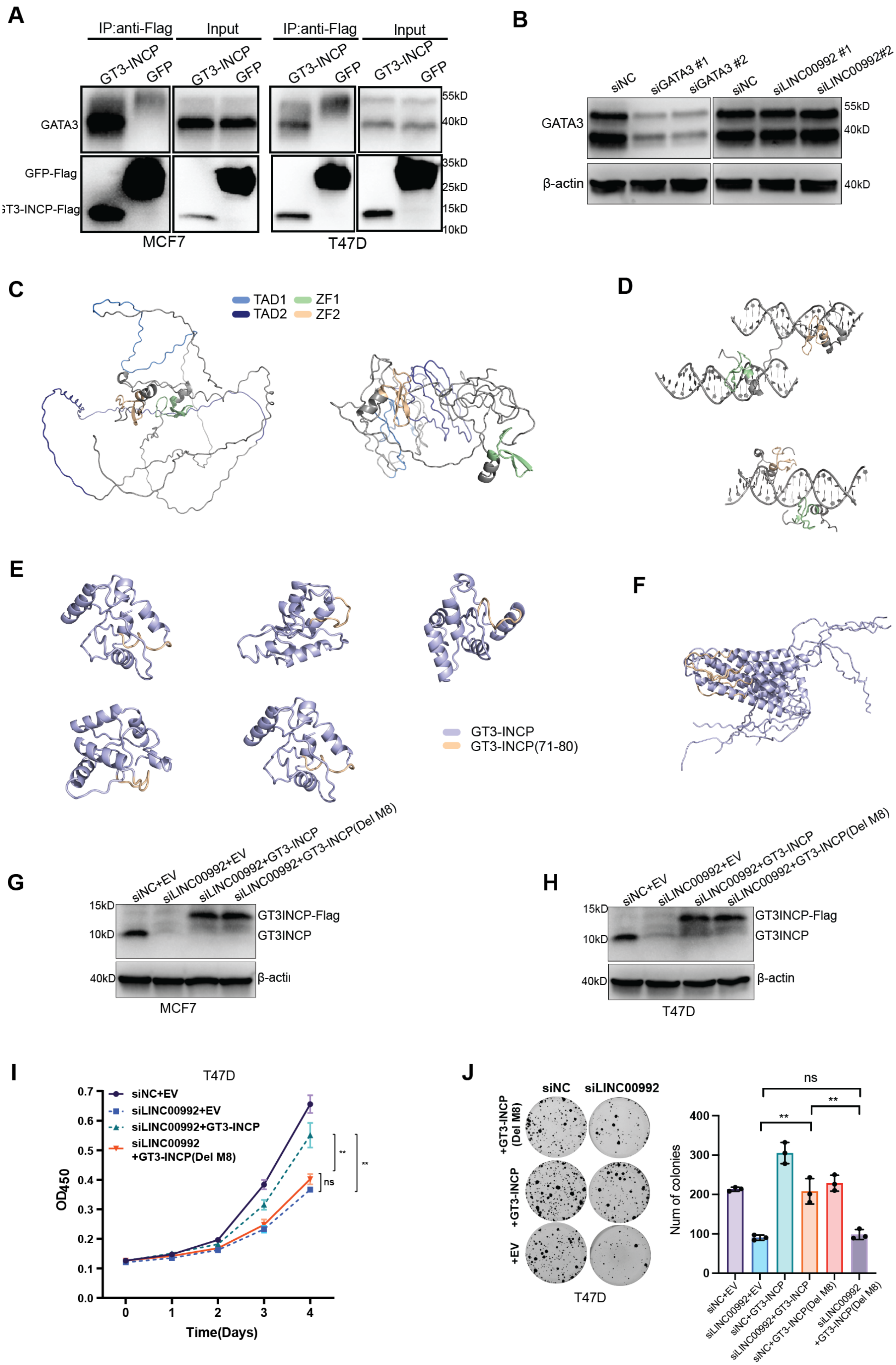

A
IP:anti-Flag
Input
GT3-INCP
GFP
GATA3
GFP-Flag
55kD
40kD
35kD
25kD
15kD
10kD
MCF7
T47D
B
siNC
siGATA3 #1
siGATA3 #2
siLINC00992 #1
siLINC00992#2
GATA3
β-actin
55kD
40kD
C
TAD1
TAD2
ZF1
ZF2
D
E
F
GT3-INCP
GT3-INCP(71-80)
G
siNC+EV
siLINC00992+EV
siLINC00992+GT3-INCP
siLINC00992+GT3-INCP(Del M8)
15kD
10kD
40kD
GT3INCP-Flag
GT3INCP
MCF7
H
β-actin
T47D
I
T47D
siNC+EV
siLINC00992+EV
siLINC00992+GT3-INCP
siLINC00992
+GT3-INCP(Del M8)
OD450
Time(Days)
ns
**
J
siNC
siLINC00992
+GT3-INCP
(Del M8)
+GT3-INCP
+EV
Num of colonies
siNC+EV
siLINC00992+EV
siNC+GT3-INCP
siLINC00992+GT3-INCP
siNC+GT3-INCP(Del M8)
siLINC00992
+GT3-INCP(Del M8)

Figure S4. (A) Whole-cell lysates of the MCF7/T47D cells stably expressing the FLAG-tagged GT3-INCP (GT3-INCP-Flag) or the negative control FLAG-tagged GFP (GFP-Flag), were immunoprecipitated (IPed) with an anti-FLAG antibody. The co-IPed proteins were then detected by the indicated antibodies. **(B)** Western blot analysis of GATA3 protein expression in the MCF7 cells transfected with the siNC or the siRNAs targeting GATA3/LINC00992. (**C)** The full-length GATA3 protein structure predicted by AlphaFold from AlphaFold Database (Left) and predicted by I-TASSER-MTD (Right) are shown. The TAD1, TAD2, ZF1 and ZF2 domain are shown in light blue, navy, green and orange, respectively. **(D)** The experimentally determined structures of ZF1/ZF2 domain from one chain GATA3 binding with two individual DNA molecules (top) and ZF1/ZF2 domain from two separate GATA3 proteins binding with one DNA molecule (bottom) (PDB ID: 4hc7). Five structural models of GT3-INCP predicted by **(E)** I-TASSER and **(F)** AlphaFold2 are shown, with the region of 71-80 aa highlighted in wheat color. Using an anti-GT3-INCP antibody, western blot analysis was performed to determine the expression of endogenous GT3-INCP and ectopic FLAG-tagged wild-type/mutant (Del M8) GT3-INCP in the **(G)** MCF7 and **(H)** T47D cells that were stably transduced with the empty vector control (EV) or the indicated ORFs, and were transfected with the siNC or a LINC00992-targeting siRNA. **(I)** The rescue of the growth defect caused by LINC00992 knockdown in T47D cells, by ectopic expression of the wild-type/ the mutant (Del-M8) GT3-INCP that loses interaction with GATA3. The T47D cells stably expressing the EV control or the indicated ORFs were transfected with the siNC/LINC00992-targeting siRNA. The cell growth was monitored by CCK-8 assay. **(J)** The rescue of the clonogenic growth defect caused by LINC00992 knockdown in T47D cells, by ectopic expression of the wild-type/the mutant (Del M8) GT3-INCP. The representative pictures of clonogenic growth and the bar graph quantifying the colonies formed by the T47D cells that were transduced with the EV or the indicated ORFs, and were transfected with the siNC or a LINC00992-targeting siRNA are shown. (**I-J**) Data are shown as mean+/-standard deviation (SD), n=3. One-way ANOVA with Tukey's multiple comparison test (**P*<0.05; ***P*<0.01; ns: not significant, *P*>0.05). (**A-B** and **G-H**) Data are representative of 3 independent experiments.

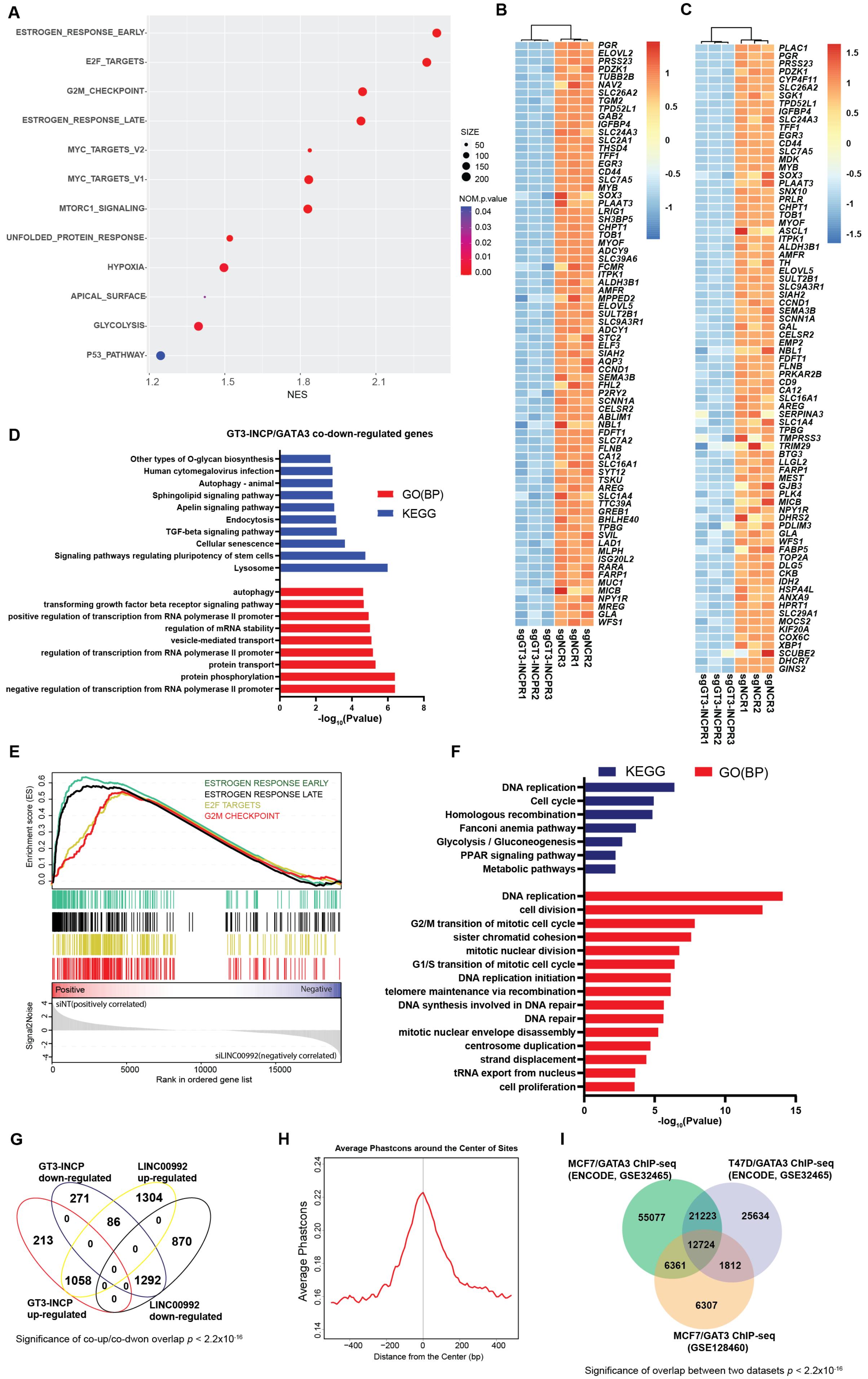
A
ESTROGEN_RESPONSE_EARLY
E2F_TARGETS
G2M_CHECKPOINT
ESTROGEN_RESPONSE_LATE
MYC_TARGETS_V2
MYC_TARGETS_V1
MTORC1_SIGNALING
UNFOLDED_PROTEIN_RESPONSE
HYPOXIA
APICAL_SURFACE
GLYCOLYSIS
P53_PATHWAY
1.2 1.5 1.8 2.1
NES
SIZE
50
100
150
200
NOM.p.value
0.04
0.03
0.02
0.01
0.00
B
PGR ELOVL2 PRSS23 PDZK1 TUBB2B NAV2 SLC26A2 TGM2 TPD52L1 GAB2 IGFBP4 SLC24A3 SLC2A1 THSD4 TFF1 EGR3 CD44 SLC7A5 MYB SOX3 PLAAT3 LRIG1 SH3BP5 CHPT1 TOB1 MYOF ADCY9 SLC39A6 FCMR ITPK1 ALDH3B1 AMFR MPPED2 ELOVL5 SULT2B1 SLC9A3R1 ADCY1 STC2 ELF3 SIAH2 AQP3 CCND1 SEMA3B FHL2 P2RY2 SCNN1A CELSR2 ABLIM1 NBL1 FDFT1 SLC7A2 FLNB CA12 SLC16A1 SYT12 TSKU AREG SLC1A4 TTC39A GREB1 BHLHE40 TPBG SVIL LAD1 MLPH ISG20L2 RARA FARP1 MUC1 MICB NPY1R MREG GLA WFS1
1
0.5
0
-0.5
-1
sgGT3-INCPR1
sgGT3-INCPR2
sgGT3-INCPR3
sgNCR3
sgNCR1
sgNCR2
C
PLAC1 PGR PRSS23 PDZK1 CYP4F11 SLC26A2 SGK1 TPD52L1 IGFBP4 SLC24A3 TFF1 EGR3 CD44 SLC7A5 MDK MYB SOX3 PLAAT3 SNX10 PRLR CHPT1 TOB1 MYOF ASCL1 ITPK1 ALDH3B1 AMFR TH ELOVL5 SULT2B1 SLC9A3R1 SIAH2 CCND1 SEMA3B SCNN1A GAL CELSR2 EMP2 NBL1 FDFT1 FLNB PRKAR2B CD9 CA12 SLC16A1 AREG SERPINA3 SLC1A4 TPBG TMPRSS3 TRIM29 BTG3 LLGL2 FARP1 MEST GJB3 PLK4 MICB NPY1R DHRS2 PDLIM3 GLA WFS1 FABP5 TOP2A DLG5 CKB IDH2 HSPA4L ANXA9 HPRT1 SLC29A1 MOCS2 KIF20A COX6C XBP1 SCUBE2 DHCR7 GINS2
1.5
1
0.5
0
-0.5
-1
-1.5
sgGT3-INCPR1
sgGT3-INCPR2
sgGT3-INCPR3
sgNCR1
sgNCR2
sgNCR3
D
GT3-INCP/GATA3 co-down-regulated genes
Other types of O-glycan biosynthesis
Human cytomegalovirus infection
Autophagy - animal
Sphingolipid signaling pathway
Apelin signaling pathway
Endocytosis
TGF-beta signaling pathway
Cellular senescence
Signaling pathways regulating pluripotency of stem cells
Lysosome
autophagy
transforming growth factor beta receptor signaling pathway
positive regulation of transcription from RNA polymerase II promoter
regulation of mRNA stability
vesicle-mediated transport
regulation of transcription from RNA polymerase II promoter
protein transport
protein phosphorylation
negative regulation of transcription from RNA polymerase II promoter
GO(BP)
KEGG
0 2 4 6 8
-log10(Pvalue)
E
Enrichment score (ES)
0.0 0.1 0.2 0.3 0.4 0.5 0.6
ESTROGEN RESPONSE EARLY
ESTROGEN RESPONSE LATE
E2F TARGETS
G2M CHECKPOINT
Positive
Negative
siNT(positively correlated)
siLINC00992(negatively correlated)
Signal2Noise
4 2 0 -2 -4
0 5000 10000 15000
Rank in ordered gene list
F
KEGG
GO(BP)
DNA replication
Cell cycle
Homologous recombination
Fanconi anemia pathway
Glycolysis / Gluconeogenesis
PPAR signaling pathway
Metabolic pathways
DNA replication
cell division
G2/M transition of mitotic cell cycle
sister chromatid cohesion
mitotic nuclear division
G1/S transition of mitotic cell cycle
DNA replication initiation
telomere maintenance via recombination
DNA synthesis involved in DNA repair
DNA repair
mitotic nuclear envelope disassembly
centrosome duplication
strand displacement
tRNA export from nucleus
cell proliferation
0 5 10 15
-log10(Pvalue)
G
GT3-INCP down-regulated
LINC00992 up-regulated
271
1304
0
86
0
213
0
0
870
0
1058
0
0
1292
0
GT3-INCP up-regulated
LINC00992 down-regulated
Significance of co-up/co-dwon overlap p < 2.2x10-16
H
Average Phastcons around the Center of Sites
Average Phastcons
0.14 0.16 0.18 0.20 0.22 0.24
-400 -200 0 200 400
Distance from the Center (bp)
I
MCF7/GATA3 ChIP-seq (ENCODE, GSE32465)
T47D/GATA3 ChIP-seq (ENCODE, GSE32465)
55077
21223
25634
12724
6361
1812
6307
MCF7/GAT3 ChIP-seq (GSE128460)
Significance of overlap between two datasets p < 2.2x10-16

Figure S5. (A) Bubble plot showing the top enriched down-regulated pathways following sgRNA-mediated GT3-INCP knockout that were identified by gene set enrichment analysis (GSEA) with the hallmark gene sets. Heatmap showing the RNA-seq based expression of the **(B)** early and **(C)** late estrogen response protein-coding genes that exhibited decreased expression in the knockout (sgGT3-INCP) group compared with the negative control (sgNC) group. **(D)** Barplot showing the top enriched Gene Ontology biological process (GO-BP) terms and KEGG pathways ranked by $-\log_{10}$(P-value), based on the functional enrichment analysis of protein-coding-genes co-down-regulated by GT3-INCP and GATA3. **(E)** GSEA with the hallmark gene sets showing the top enriched gene sets down-regulated by siRNA-mediated LINC00992 knockdown. **(F)** Barplot showing the top enriched GO-BP terms and KEGG pathways ranked by $-\log_{10}$(P-value), based on the functional enrichment analysis of the protein-coding-genes down-regulated by siRNA-mediated knockdown of LINC00992 and GATA3. **(G)** Venn diagram showing the overlaps of the differentially expressed protein-coding genes that were identified from RNA-seq data, upon siRNA-mediated LINC00992 knockdown or sgRNA-mediated GT3-INCP knockout. (**H**) The Conservation plot showing that the GT3-INCP binding sites identified from ChIP-seq data have higher PhastCons scores (i.e, are more conserved) than their flanking regions. **(I)** Venn diagram showing the overlaps of the GATA3 binding sites in the MCF7/T47D cells among three ChIP-seq datasets (GSE32465 and GSE128460). Fisher's exact test was used to assess the statistical significance of the venn diagram overlap (**G** and **I**).

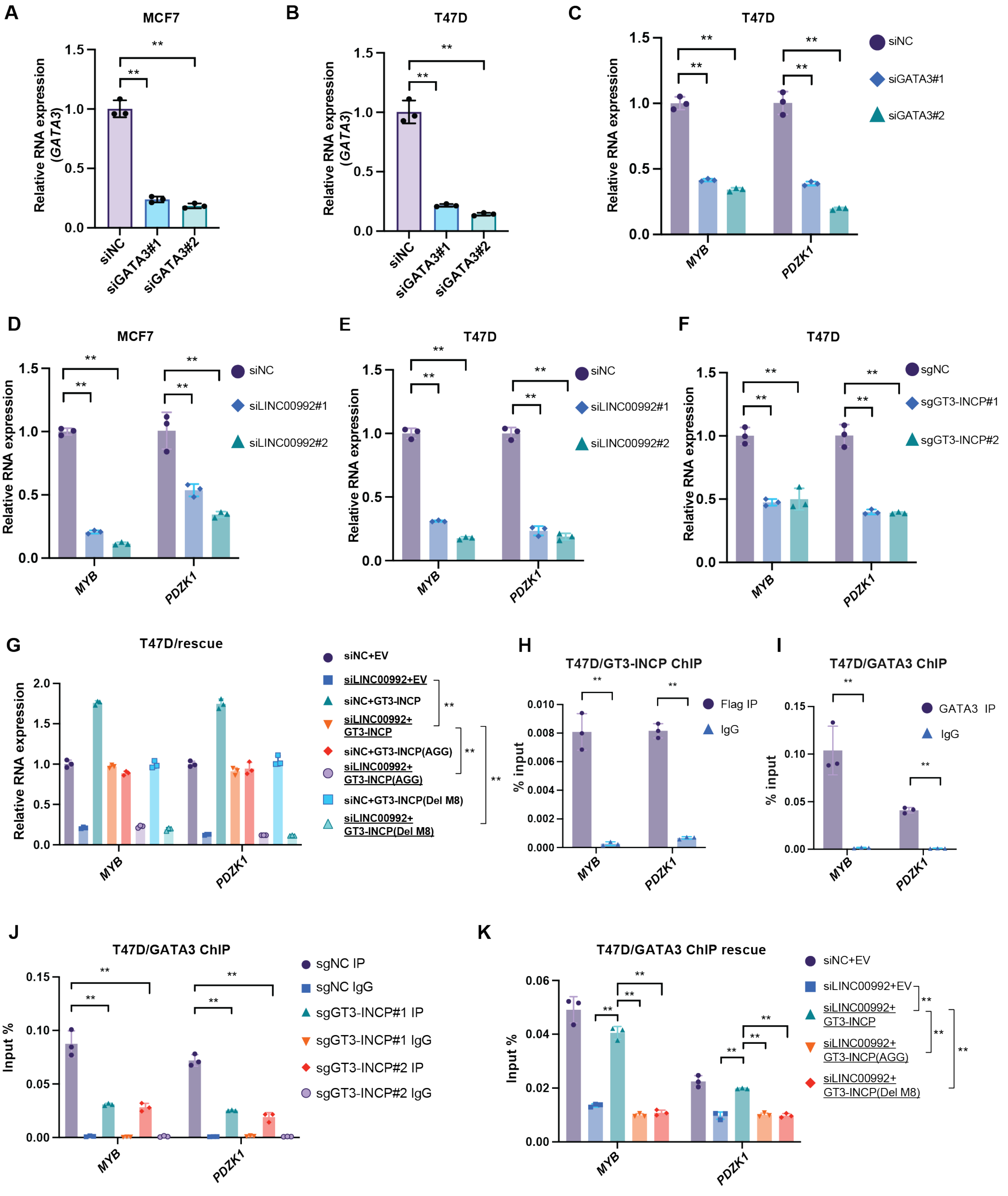
A
MCF7
Relative RNA expression (GATA3)
siNC
siGATA3#1
siGATA3#2
B
T47D
C
T47D
Relative RNA expression
MYB
PDZK1
siNC
siGATA3#1
siGATA3#2
D
MCF7
siNC
siLINC00992#1
siLINC00992#2
E
T47D
F
T47D
sgNC
sgGT3-INCP#1
sgGT3-INCP#2
G
T47D/rescue
siNC+EV
siLINC00992+EV
siNC+GT3-INCP
siLINC00992+ GT3-INCP
siNC+GT3-INCP(AGG)
siLINC00992+ GT3-INCP(AGG)
siNC+GT3-INCP(Del M8)
siLINC00992+ GT3-INCP(Del M8)
H
T47D/GT3-INCP ChIP
% input
Flag IP
IgG
I
T47D/GATA3 ChIP
GATA3 IP
IgG
J
T47D/GATA3 ChIP
Input %
sgNC IP
sgNC IgG
sgGT3-INCP#1 IP
sgGT3-INCP#1 IgG
sgGT3-INCP#2 IP
sgGT3-INCP#2 IgG
K
T47D/GATA3 ChIP rescue
siNC+EV
siLINC00992+EV
siLINC00992+ GT3-INCP
siLINC00992+ GT3-INCP(AGG)
siLINC00992+ GT3-INCP(Del M8)

Figure S6. QRT-PCR was performed to determine the siRNA-mediated GATA3 knockdown efficiency in **(A)** MCF7 and **(B)** T47D cells. QRT-qPCR analysis of the MYB and PDZK1 RNA expression change following (**C**) siRNA-mediated GATA3 knockdown in T47D cells, siRNA-mediated LINC00992 knockdown in (**D**) MCF7 and (**E**) T47D cells, and (**F**) sgRNA-mediated GT3-INCP knockout in T47D cells. **(G)** In the presence of LINC00992 knockdown, the rescue effect of ectopic expression of the wild-type/mutant GT3-INCP (Del-M8 or AGG mutation in start codon) with respect to the empty vector control (EV), on the MYB and PDZK1 RNA expression, was assessed by qRT-PCR analysis in T47D cells. **(H)** ChIP-qPCR analysis was performed with an anti-FLAG/anti-IgG antibody in the T47D cells stably expressing the FLAG-tagged GT3-INCP to determine the binding of GT3-INCP to the ChIP-seq identified common GATA3 binding sites around *MYB* and *PDZK1* **(I)** ChIP-qPCR analysis was performed with an anti-GATA3/anti-IgG antibody in T47D cells to determine the occupancy of GATA3 on the ChIP-seq identified GATA3 binding sites around *MYB* and *PDZK1*. **(J)** ChIP-qPCR analysis for assessing the effect of GT3-INCP knockout on GATA3 occupancy on its own binding sites around *MYB* and *PDZK1* in T47D cells. **(K)** In the presence of LINC00992 knockdown, ChIP-qPCR analysis was performed to assess the rescue effect of ectopic expression of the wild-type or mutant GT3-INCP (Del-M8 or AGG) with respect to the EV control, on the occupancy of GATA3 on its binding sites around *MYB* and *PDZK1* in T47D cells. (**A**-**K**) Data are shown as mean+/-standard deviation (SD), n=3. (**H**-**I**) Two-tailed unpaired Student's t-test (**P*<0.05; ***P*<0.01; ns: not significant, *P*>0.05). (**A**-**G** and **J**-**K**) One-way ANOVA with Dunnett's multiple comparison test (**P*<0.05; ***P*<0.01; ns: not significant, *P*>0.05).

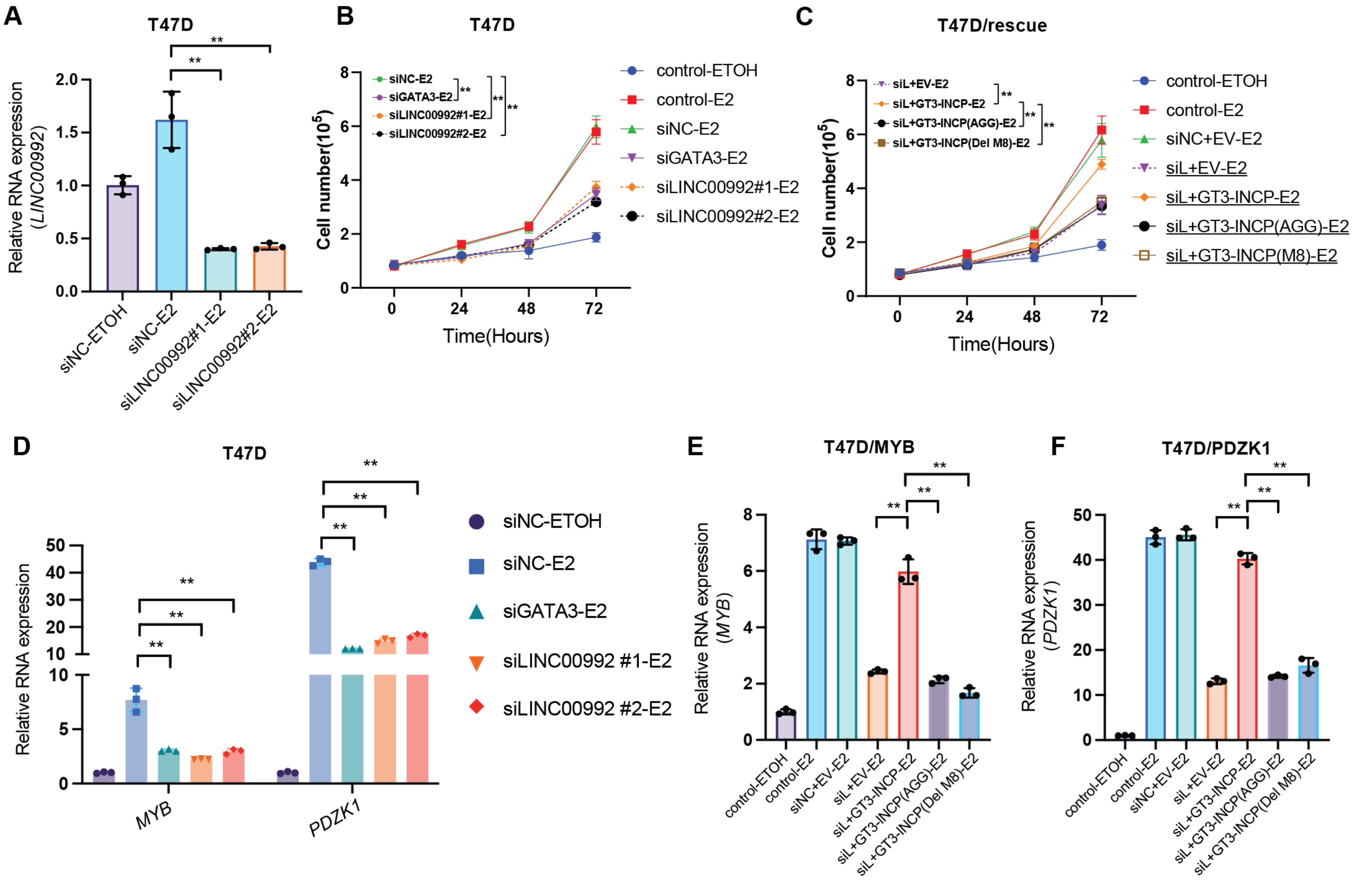

Figure S7. (**A**) qRT-PCR analysis of LINC00992 RNA expression in the T47D cells that were transfected with the negative control siRNA (siNC) or LINC00992-targeting siRNAs (siLINC00992), after E2 (30 nM) or ETOH vehicle treatment (ETOH). **(B)** After E2/ETOH.treatment, the numbers of the T47D cells treated with the transfection reagent (control) or transfected with the siNC, GATA3-targeting siRNAs (siGATA3) or siLINC00992 were counted every 24 hs for 72 hs. **(C)** After E2/ETOH treatment, the number of the T47D cells that were treated with the transfection reagent (control) or the T47D cells that were transduced with the EV or the indicated ORFs and were transfected with the siNC/siLINC00992 (siL), was counted every 24 hs for 72 hs. **(D)** qRT-PCR analysis of the MYB and PDZK1 RNA expression in the MCF7 cells that were transfected with the siNC, siGATA3 or siLINC00992, after E2/ETOH treatment. QRT-PCR analysis of the (**E**) MYB and (**F**) PDZK1 RNA expression in the T47D cells that were treated with the transfection reagent (control) or the T47D cells that were transduced with the EV or the indicated ORFs and were transfected with the siNC/LINC00992-targeting siRNA (siL), after E2/ETOH treatment. (**A-F**) Data are shown as mean+/-standard deviation (SD), n=3. One-way ANOVA with Dunnett's multiple comparison test (**P*<0.05; ***P*<0.01; ns: not significant, *P*>0.05).

.
